# Quantized State Estimation for Linear Dynamical Systems

**DOI:** 10.3390/s24196381

**Published:** 2024-10-01

**Authors:** Ramchander Rao Bhaskara, Manoranjan Majji, Felipe Guzmán

**Affiliations:** 1 Department of Aerospace Engineering, Texas A&M University, College Station, TX 77843, USA; mmajji@tamu.edu; 2 Wyant College of Optical Sciences, The University of Arizona, Tucson, AZ 85721, USA; felipeguzman@arizona.edu

**Keywords:** optical sensors, Kalman filter, state estimation, quantized filtering, finite-precision, FPGA

## Abstract

This paper investigates state estimation methods for dynamical systems when model evaluations are performed on resource-constrained embedded systems with finite precision compute elements. Minimum mean square estimation algorithms are reformulated to incorporate finite-precision numerical errors in states, inputs, and measurements. Quantized versions of least squares batch estimation, sequential Kalman, and square-root filtering algorithms are proposed for fixed-point implementations. Numerical simulations are used to demonstrate performance improvements over standard filter formulations. Steady-state covariance analysis is employed to capture the performance trade-offs with numerical precision, providing insights into the best possible filter accuracy achievable for a given numerical representation. A low-latency fixed-point acceleration state estimation architecture for optomechanical sensing applications is realized on Field Programmable Gate Array System on Chip (FPGA-SoC) hardware. The hardware implementation results of the estimator are compared with double-precision MATLAB implementation, and the performance metrics are reported. Simulations and the experimental results underscore the significance of modeling quantization errors into state estimation pipelines for fixed-point embedded implementations.

## 1. Introduction

Kalman filters were first introduced into onboard guidance and navigation systems during NASA’s Apollo Project in the 1960s [1]. However, digital simulations of the filter for onboard trajectory estimation using finite-precision arithmetic uncovered numerical stability problems. The filter simulations on the IBM 704 computer with 36-bit floating-point arithmetic were determined to be numerically unreliable. On the Apollo flight computer constrained with only 15-bit fixed-point arithmetic operations, onboard implementation of the filter was infeasible [2]. Since then, efforts to improve filter accuracy and stability under finite-precision hardware constraints have been a focus for practical realization of navigation filters. Square-root filtering was invented as a solution for execution on the Apollo guidance computer to solve the historic circumlunar navigation problem [3]. Square-root algorithms for state estimation were well-established by the 1970s [4,5] to address numerical stability issues due to quantization effects. With technological advancements in microprocessors and hardware accelerators, research on navigation filters continued to focus on high-speed, hard real-time calculations [6]. Recent research on quantized filtering algorithms has been enabled by optimized software packages, dedicated hardware for parallel computing, and purpose-built solutions [7,8,9].

The quantization problem in state estimation and optimal control has been widely studied in the literature [10,11,12]. Quantization is commonly utilized in wireless sensor networks (WSNs) with low-quality sensors with limited computation and communication capabilities, and where transmission bandwidth is severely constrained. A class of Kalman filters where the difference between measurements and its predictions (i.e., innovations) are quantized has been developed for decentralized state estimation [12,13]. In these quantized Kalman filters, measurement predictions are distributed from a fusion center to a sensor node, where innovations are quantized and sent back to the center for filter update. The sign of innovation Kalman filter (SOI-KF) [13] operates by quantizing innovations to only 1-bit, which may give rise to large estimation errors. Filters are also proposed for multi-bit transmission, such as the multiple-level quantized innovation Kalman filter (MLQ-KF) [12]. It is shown that with multi-bit quantization, the performance of the quantized filter can be recovered to nearly match the mean squared errors of standard the Kalman filter [12]. However, to have such performance, uniformly distributed sensor nodes collectively convey their quantized information to a fusion center for processing into a combined target state estimate. Having just one isolated sensor significantly undermines estimator tracking performance, and a standard Kalman filter operating on high-resolution measurements from even one sensor is relatively more accurate. Work has also been reported in utilizing quantized measurements in the development and analysis of particle filters [14], unscented Kalman filters [15], and their fixed-point implementations [14,16]. These implementations are purpose-built for accelerating filter algorithms for specific applications and are not easily scalable for generic state estimation problems. To a great extent, the usual practice for high-speed onboard realization of filtering algorithms vastly depends upon the hardware resources and also on the choice of an optimal state estimator. This paper aims to bridge this gap by reformulating minimum mean square estimation algorithms to explicitly account for finite-precision numerical errors in states, inputs, and measurements. By integrating quantization noise models into the filter structure, the proposed approach enables the design of quantized filters that are better suited for fixed-point implementations on embedded systems.

This research focuses on optimal quantized filtering methods with an application focus on optomechanical acceleration sensors for inertial navigation. Optomechanical accelerometers rely on the coupling between the mechanical displacement of a test mass and light captured using an optical detection system [17]. Such a sensor requires optical data processing on high-speed hardware modules such as Field Programmable Gate Arrays (FPGAs) [18] for estimation of acceleration forces from test-mass displacement measurements. These precision force measuring units are deployed in geodesic applications, including the Gravity Recovery and Climate Experiment (GRACE) mission [19], the Laser Interferometer Space Antenna (LISA) [20], and the Laser Interferometer Gravitational-Wave Observatory (LIGO) [21].

For deployment on a spacecraft or resource-constrained hardware (e.g., FPGA [22,23]), dynamic range and sensor resolution limitations shall restrict the ability of standard filtering algorithms to precisely estimate states, especially when the dynamical system exhibits large variations in state variables. This necessitates the adoption of accurate error models and the use of novel filtering techniques to achieve sufficient estimation accuracy of desired state variables from the sensor measurements [10,12,24]. Furthermore, in scenarios where transmitting high-resolution measurements or performing double-precision operations is impossible or memory-intensive, quantization errors become significant. With increased performance requirements, the noise from the quantization effects has become an important aspect to analyze in efforts to maximize signal-to-noise ratio [12,13,25,26]. For the state estimation problem of dynamical stochastic processes, finite-precision implementation necessitates estimation to be based on quantized parameters of state, input, and observations. This requires revisiting the state estimation algorithms to include quantization errors in the filter implementation. Although using a sufficiently large word length for real-valued state variables can minimize the effects of quantization, even with a large number of bits, the system cannot be completely detached from finite word-length effects. In some instances, the luxury of large dynamic range for storage and computations may not be practicable. Consequently, the errors due to quantization have to be modeled as process and output noises, which can be accounted for in the system design using classical state-space and estimator modeling schemes.

The key contributions of this work are briefly summarized as follows. Given a finite-precision representation for system variables, linear state estimation filters are proposed. They consist of a (1) minimum variance estimator (least squares), quantized forms of (2) a discrete-time Kalman filter (QDKF), and (3) a square-root Kalman filter (QSRKF). The filter algorithms are applied to estimate states of an optomechanical oscillator model. The model formulation and assumptions are based upon the efforts of Kelly et al. [27] and the bench top experiments from Hines et al. [28]. Numerical simulations of the estimators are reported to show the prominence of modeling quantization errors in state estimation filters. The results offer much improved estimator performances over standard implementations of the respective filters when system variables are quantized. Steady-state filter performance is analyzed to provide insights into the best possible filter accuracy achievable for a given numerical representation. A least squares-based dual oscillator model is implemented on the FPGA board for the state estimation of acceleration forces from simulated measurements. A reliable estimator performance is reported by comparing the fixed-point implementation on the FPGA with the floating-point software implementation as a reference. Note that, in this work, quantization noise is from rounding off of numerical data to a desired number of bits. From here on, quantization errors and round-off errors are used interchangeably and refer to the same idea. Moreover, bit overflows are assumed to be negligible due to the careful selection of dynamic ranges for internal variable representation.

The rest of the article is organized as follows. The sensor model is described in Section 2. The quantization effects are incorporated into the model and state estimation filters are proposed in Section 3. Respective filter algorithms are derived in Appendix A, Appendix B and Appendix C. Numerical simulations are reported in Section 4 to illustrate the performance of the proposed filter structures. In Section 5, an FPGA architecture is proposed for on-board implementation of a dual-oscillator filter for acceleration estimation problem. Implementation results are reported. Concluding remarks are drawn in Section 6.

## 2. Description of Sensor Dynamics

The quantized filter performances are investigated for state estimation of the dynamical system described in this section. The generalized filtering algorithms are developed in Appendix A, Appendix B and Appendix C.

### 2.1. Discrete-Time Sensor Model

The one degree-of-freedom (1-DOF) accelerometer sensor dynamics are modeled as a second order spring-mass-damper system [29], equivalent to a perturbed linear harmonic oscillator. This formulation describes a direct conversion accelerometer, where the displacement of the proof mass x(t) is directly measured using precise laser wavelength as a length reference [30]. Accounting for drift-causing optical and thermomechanical noise sources, through a white noise process and a biasing term, the dynamics for the proof mass displacement in response to the single-axis forcing function g(t) are formulated using the following equations developed by Kelly [27] et al.:

(1) $$\begin{array}{cc} \ddot{x}+2\omega \zeta \dot{x}+\omega^{2}x & =g\left(t\right)+b\left(t\right)+n_{v}\left(t\right) \end{array}$$

(2) $$\begin{array}{cc} \dot{b}\left(t\right) & =n_{u}\left(t\right) \end{array}$$

where ω is the natural frequency of the oscillator, ζ is its damping factor, and the bias term b(t) is modeled as a Wiener process. In discrete time, the increments of this Wiener process can be represented as an independent and identically distributed Gaussian random sequence. The terms $n_{v}\left(t\right)$ and $n_{u}\left(t\right)$ are uncorrelated, zero-mean Gaussian white-noise processes with spectral densities $\sigma_{v}^{2}$ and $\sigma_{u}^{2}$, respectively.

In continuous-time state space description, the 1-DOF accelerometer model is

(3) $$\begin{array}{c} \dot{X}\left(t\right)=\left[\begin{array}{ccc} 0 & 1 & 0\\ -\omega^{2} & -2\omega \zeta & 1\\ 0 & 0 & 0 \end{array}\right]X\left(t\right)+\left[\begin{array}{c} 0\\ 1\\ 0 \end{array}\right]g\left(t\right)+\left[\begin{array}{c} 0\\ n_{v}\left(t\right)\\ n_{u}\left(t\right) \end{array}\right] \end{array}$$

(4) $$\begin{array}{c} y\left(t\right)=\left[\begin{array}{ccc} 1 & 0 & 0 \end{array}\right]X\left(t\right)+\nu \left(t\right) \end{array}$$

where the states of the system are $X\left(t\right)={\left[x\left(t\right)\;\dot{x}\left(t\right)\;b\left(t\right)\right]}^{T}$ representing displacement, velocity, and bias, in that order. The measurement model implicitly assumes that the position state, x(t), is observable and that the sensor dynamics do not consider off-axis accelerations.

The linear system in Equation (3) can be discretized using a zero-order hold (ZOH) approximation, assuming that inputs and noise change only at discrete sampling intervals Δt. In this discretization, $g_{k}$ is assumed constant over each sampling interval. However, it’s crucial to note that if $g_{k}$ varies more rapidly than can be observed within the interval Δt, the Nyquist theorem limits our ability to estimate $g_{k}$ accurately. This highlights the importance of selecting an appropriate sampling rate in relation to the system’s dynamics. The discretized state space model is

(5) $$\begin{array}{c} X_{k+1}=\Phi \left(t_{k+1},t_{k}\right)X_{k}+\Gamma \left(t_{k+1},t_{k}\right)g_{k}+w_{k} \end{array}$$

where the transition from a state $X_{k+1}$ to $X_{k}$ for the linear time-invariant system is determined by a matrix exponential of the system matrix in Equation (3) (denoted as *A*):

(6) $$\begin{array}{c} \Phi \left(t_{k+1},t_{k}\right)=e^{A\Delta t} \end{array}$$

Also,

(7) $$\begin{array}{c} \begin{array}{cc} \Psi (t_{k+1},t_{k}) & =\int_{t_{k}}^{t_{k+1}}\Phi \left(t_{k+1},\tau \right)d\tau \\ \Gamma (t_{k+1},t_{k}) & ={\left[\begin{array}{ccc} \Psi_{12}\left(t_{k+1},t_{k}\right) & \Psi_{22}\left(t_{k+1},t_{k}\right) & \Psi_{32}\left(t_{k+1},t_{k}\right) \end{array}\right]}^{T} \end{array} \end{array}$$

The stochastic process noise term $w_{k}$, additive to the discrete-time state evolution, is described as

(8) $$\begin{array}{c} \begin{array}{cc} w_{k} & =\int_{t_{k}}^{t_{k+1}}\Phi \left(t_{k+1},\tau \right){\left[\begin{array}{ccc} 0 & n_{v}\left(\tau \right) & n_{u}\left(\tau \right) \end{array}\right]}^{T}d\tau \\ Q & =E[w_{k}w_{k}^{T}] \end{array} \end{array}$$

For the linear system described here, the state transition matrix Φ and the covariance matrix Q associated with $w_{k}$ can both be analytically derived [27]. E[·] denotes the expected value operator.

Regarding the observation model, the displacement measurements are acquired at a fixed rate from an accurate one-dimensional interferometric displacement sensor. Following the state-space representation in Equation (4), the discrete-time measurement model is given by

(9) $$y_{k}=x_{k}+\nu_{k}$$

This measurement model embeds the analog readout noise $\nu_{k}$ in the measurements. This observation error is treated as zero-mean white noise with an error variance of $\sigma_{m}^{2}$.

However, in practice, a change in the sensor’s output does not always translate exactly to a change in the mechanical input. As a result, the one-to-one association between the measured and the physical displacements in Equation (27) may not always hold true. The deviation in the sensor’s sensitivity is modeled through a scale factor and is estimated through sensor calibration and compensated during device operation [31]. The process of scale factor calibration and its associated uncertainties are not addressed in this paper.

However, the scale factor error arising from calibration uncertainty can be incorporated into the readout noise model for state estimation. To this extent, departure from unity scaling is modeled through scale factor error $\epsilon_{s}$ to account for the total readout error:

(10) $$\begin{array}{c} y_{k}=\left(1+\epsilon_{s,k}\right)x_{k}+\nu_{k} \end{array}$$

Although not rigorously treated, this direction is briefly noted in Section 3.3.

### 2.2. Calibrated Sensor Model

Accelerometer bias accumulates over time, as indicated in Equation (2). The bias model is

(11) $$b_{k+1}=b_{k}+\int_{t_{k}}^{t_{k+1}}n_{u}\left(\tau \right)d\tau$$

A reasonable calibration step must be implemented periodically to correct for the sensor bias to prevent its build-up effect on state estimation. A calibration sequence shall be performed by applying a known input to the sensor and observing the system response (as described in previous work [27]). This calibration step provides us with an estimate of the bias $\hat{b}$ and its error statistics as follows:

(12) $$\begin{array}{cc} \; & b_{0}=\hat{b}+n_{b_{0}}\;\mathrm{and}\;b_{k}=b_{0}+\int_{t_{0}}^{t_{k}}n_{u}\left(\tau \right)d\tau \end{array}$$

(13) $$\begin{array}{cc} \; & E[b_{k}-\hat{b}]=0 \end{array}$$

(14) $$\begin{array}{cc} \; & E\left[\left(b_{k}-\hat{b}\right){\left(b_{k}-\hat{b}\right)}^{T}\right]=\sigma_{b0}^{2}+\left(t_{k}-t_{0}\right)\sigma_{u}^{2} \end{array}$$

where, at the time of calibration $t_{0}$, the unbiased bias estimate $b_{0}$ is corrupted by a white noise source $n_{b0}$ and has a variance of $\sigma_{b0}^{2}$. The bias estimate is assumed to not significantly degrade between periodic calibrations, but its variance grows as the bias evolves with a dependence upon the zero-mean Gaussian process $n_{u}\left(t\right)$ as shown in Equation (2).

Assuming that an independent calibration step has been performed to estimate the sensor bias, the model dynamics in position and velocity (Equation (5)), influenced by the instantaneous bias term $b_{k}$, can be written as follows:

(15) $$\begin{array}{c} \begin{array}{cc} \; & \left[\begin{array}{c} x_{k+1}\\ {\dot{x}}_{k+1} \end{array}\right]=\left[\begin{array}{cc} \Phi_{11} & \Phi_{12}\\ \Phi_{21} & \Phi_{22} \end{array}\right]\left[\begin{array}{c} x_{k}\\ {\dot{x}}_{k} \end{array}\right]+\left[\begin{array}{c} \Psi_{12}\\ \Psi_{22} \end{array}\right]g_{k}+\left[\begin{array}{c} \Phi_{13}\\ \Phi_{23} \end{array}\right]\hat{b}+{\tilde{w}}_{k}\\ & {\tilde{w}}_{k}=\left[\begin{array}{c} \Phi_{13}\\ \Phi_{23} \end{array}\right]\left(b_{k}-\hat{b}\right)+\int_{t_{k}}^{t_{k+1}}\left[\begin{array}{c} n_{v}\left(\tau \right)\Phi_{12}\left(t_{k+1},\tau \right)\\ n_{v}\left(\tau \right)\Phi_{22}\left(t_{k+1},\tau \right) \end{array}\right]d\tau \end{array} \end{array}$$

It can be shown that, to integrate the uncertainty in the instantaneous bias value into the system dynamics, the redefined process noise covariance $\tilde{Q}$ is derived as:

(16) $$\begin{array}{c} \begin{array}{cc} \tilde{Q}\; & =\left[\begin{array}{cc} \Phi_{13}^{2} & \Phi_{13}\Phi_{23}\\ \Phi_{23}\Phi_{13} & \Phi_{23}^{2} \end{array}\right]\left(\sigma_{b0}^{2}+\sigma_{u}^{2}\left(t_{k}-t_{0}\right)\right)+\\ \; & \sigma_{v}^{2}\left[\begin{array}{cc} \int_{t_{k}}^{t_{k+1}}\Phi_{12}^{2}\left(t_{k+1},\tau \right)d\tau & \int_{t_{k}}^{t_{k+1}}\Phi_{12}\left(t_{k+1},\tau \right)\Phi_{22}\left(t_{k+1},\tau \right)d\tau \\ \int_{t_{k}}^{t_{k+1}}\Phi_{12}\left(t_{k+1},\tau \right)\Phi_{22}\left(t_{k+1},\tau \right)d\tau \; & \int_{t_{k}}^{t_{k+1}}\Phi_{22}^{2}\left(t_{k+1},\tau \right)d\tau \end{array}\right] \end{array} \end{array}$$

The objective of this work is to estimate the forcing acceleration from position measurements. To achieve this, the governing equations for the system dynamics can be leveraged to estimate the states using either a minimum variance or a Kalman filter approach. However, the practical implementation of an onboard state estimation filter also accounts for additional artifacts arising from implementation on finite-precision computing architecture. In particular, discrete-time systems are susceptible to numerical errors when finite word-length registers are used to represent the states and measurements [25]. For accurate estimation of the forcing acceleration, it is essential to account for these finite word-length effects due to quantization. Neglecting such effects will degrade the estimation accuracy, as will be shown through the numerical simulations that follow. Thus, quantization effects necessitate reformulating the state-space model described in Equation (5) to account for the corresponding numerical errors.

## 3. Model Reformulation and State Estimation

### 3.1. Dynamical System: Fixed-Point Realization

A fixed-point realization of a discrete-time system is a problem that considers the presence of quantization noise due to rounding off of products within the realization. Systematic approaches to deal with the adverse effects of fixed-point implementation in digital filters are developed by Mullis [26], Hwang [32], Williamson and Kadiman [33], Liu and Skelton [10]. These approaches involve formulating discrete-time models, such as those in Equations (5) and (9) where the system state $X_{k}$, input $g_{k}$, and the measurement variable $y_{k}$ are quantized at each instance of computation. The discrete-time model in Equation (5) is now redefined as

(17) $$\begin{array}{c} X_{k+1}=\Phi \left(t_{k+1},t_{k}\right)Q\left[X_{k}\right]+\Gamma \left(t_{k+1},t_{k}\right)Q\left[g_{k}\right]+w_{k} \end{array}$$

(18) $$\begin{array}{c} y_{k}=\left[1\;0\;0\right]Q\left[X_{k}\right]+\nu_{k} \end{array}$$

where Q[.] here represents the quantization by round-off. The additive property of the round-off errors enables their modeling into the system description as

(19) $$\begin{array}{cc} \; & Q\left[X_{k}\right]=X_{k}+\epsilon_{x,k}\;\left(\mathrm{state}\;\mathrm{quantization}\right) \end{array}$$

(20) $$\begin{array}{cc} \; & Q\left[g_{k}\right]=g_{k}+\epsilon_{g,k}\;\left(\;D/A\;\mathrm{conversion}\right) \end{array}$$

(21) $$\begin{array}{cc} \; & Q\left[y_{k}\right]=y_{k}+\epsilon_{y,k}\;\left(\;A/D\;\mathrm{conversion}\right) \end{array}$$

Here, $\epsilon_{x,k}$ arises from quantization at the state nodes, $\epsilon_{g,k}$ is from digital-to-analog (D/A) conversion of input, and $\epsilon_{y,k}$ stems from rounding-off of the sampled measurements from an analog-to-digital (A/D) converter. This approach assumes that the state nodes are quantized after double-length accumulation. However, round-off errors in the coefficients are not independently treated in this model. It is assumed that coefficient round-off errors accumulate at the state nodes, and optimizing for state quantization tends to also account for coefficient quantization errors, as direct optimization of coefficient errors is not tractable [10].

Typically, round-off errors are characterized as zero-mean independent random variables that follow a uniform distribution [34]. This modeling approach accurately captures the inherent uncertainty associated with the precision of numerical computations. Therefore, the error statistics of round-off errors can be described as

(22) $$\begin{array}{cc} \; & E\left\{\epsilon_{x,k}\right\}=0\;\;\forall \;\;k\;\mathrm{and}\;\Sigma_{x}=E\left\{\epsilon_{x,k}\epsilon_{x,k}^{T}\right\}=q_{x}I_{x};\;q_{x}≜\frac{2^{-2B_{x}}}{12} \end{array}$$

(23) $$\begin{array}{cc} \; & E\left\{\epsilon_{g,k}\right\}=0\;\;\forall \;\;k\;\mathrm{and}\;\Sigma_{g}=E\left\{\epsilon_{g,k}\epsilon_{g,k}^{T}\right\}=q_{g};\;q_{q}≜\frac{2^{-2B_{g}}}{12} \end{array}$$

(24) $$\begin{array}{cc} \; & E\left\{\epsilon_{y,k}\right\}=0\;\;\forall \;\;k\;\mathrm{and}\;\Sigma_{y}=E\left\{\epsilon_{y,k}\epsilon_{y,k}^{T}\right\}=q_{y};\;q_{y}≜\frac{2^{-2B_{y}}}{12} \end{array}$$

where $B_{x}$, $B_{g}$, and $B_{y}$ represent the word-lengths of state node registers and A/D and D/A converters. $I_{X}$ is an identity matrix corresponding to the number of states. For a multi-output system where multiple measurements are available at an epoch, the measurement round-off covariance is $qI_{y}$, as will be utilized in the subsequent derivation of the least squares estimator. By extension, for a multi-input system, the input round-off covariance could be defined as $qI_{g}$. $I_{y}$ and $I_{g}$ have the dimensions that correspond to the number of inputs and outputs. The multi-input multi-output generalization is provided in Appendix A, Appendix B and Appendix C.

### 3.2. Formulation of Least Squares Estimator

The linear time-invariant system in Equation (15) implies that a measurement obtained at the nth instant from *k* (n>k) is related to the states and input at the instant $t_{k}$ as

(25) $$\begin{array}{c} y_{k+n}=\left[\begin{array}{ccc} \Phi_{11}^{\left(n\right)} & \Phi_{12}^{\left(n\right)} & \Psi_{12}^{\left(n\right)} \end{array}\right]\left[\begin{array}{c} x_{k}\\ {\dot{x}}_{k}\\ g_{k} \end{array}\right]+\Phi_{13}^{\left(n\right)}\hat{b}+{\tilde{\nu }}_{k+n} \end{array}$$

(26) $$\begin{array}{c} {\tilde{v}}_{k+n}=\nu_{k+n}+\Phi_{13}^{\left(n\right)}\left(b_{k}-\hat{b}\right)+\int_{t_{k}}^{t_{k+n}}\Phi_{12}\left(t_{k+n},\tau \right)n_{v}d\tau \end{array}$$

where $\Phi_{ij}^{\left(n\right)}$ and $\Psi_{ij}^{\left(n\right)}$ represent the respective elements of $\Phi (t_{k+n},t_{k})$ and $\Psi (t_{k+n},t_{k})$ (refer Equation (5)).

For a batch of N+1 position measurements during which the input acceleration and the bias are assumed unchanged, the instantaneous states and the acceleration input are linearly related as follows:

(27) $$\begin{array}{c} \underset{\tilde{y}}{\underset{︸}{\left[\begin{array}{c} y_{k}\\ y_{k+1}\\ \vdots \\ y_{k+N} \end{array}\right]}}=\underset{H_{k}}{\underset{︸}{\left[\begin{array}{ccc} 1 & 0 & 0\\ \Phi_{11}^{\left(1\right)} & \Phi_{12}^{\left(1\right)} & \Psi_{12}^{\left(1\right)}\\ & \vdots & \\ \Phi_{11}^{\left(N\right)} & \Phi_{12}^{\left(N\right)} & \Psi_{12}^{\left(N\right)} \end{array}\right]}}\underset{{\tilde{X}}_{k}}{\underset{︸}{\left[\begin{array}{c} x_{k}\\ {\dot{x}}_{k}\\ g_{k} \end{array}\right]}}+\underset{\eta_{k}}{\underset{︸}{\left[\begin{array}{c} 0\\ \Phi_{13}^{\left(1\right)}\\ \vdots \\ \Phi_{13}^{\left(N\right)} \end{array}\right]}}\hat{b}+\underset{{\tilde{\nu }}_{k}}{\underset{︸}{\left[\begin{array}{c} {\tilde{\nu }}_{k}\\ {\tilde{\nu }}_{k+1}\\ \vdots \\ {\tilde{\nu }}_{k+N} \end{array}\right]}} \end{array}$$

where the forcing acceleration input $g_{k}$ is now treated as an additional state for estimation.

Additionally, incorporating round-off errors described in Equation (17) into the above model yields:

(28) $$\begin{array}{c} \tilde{y}=H_{k}\left({\tilde{X}}_{k}+\epsilon_{\tilde{X},k}\right)+\eta_{k}\left(\hat{b}+\epsilon_{\hat{b},k}\right)+{\tilde{\nu }}_{k}+\epsilon_{\tilde{y}} \end{array}$$

where the bias round-off error ($\epsilon_{\hat{b},k}$) is assumed to have the same variance as that of state ($q_{x}$). The input round-off error from D/A, $\epsilon_{g,k}$, is absorbed into the estimation state error $\epsilon_{\tilde{X},k}$. Finally, the uncorrelated errors due to quantization in the measurement batch are accumulated in a vector of length N+1 as $\epsilon_{\tilde{y}}$.

Grouping the error terms together in a new variable μ, the above linear system of equations can be expressed as

(29) $$\begin{array}{c} \begin{array}{cc} \; & \tilde{y}-\eta_{k}\hat{b}=H_{k}{\tilde{X}}_{k}+\mu \\ \; & \mu =H_{k}\epsilon_{\tilde{X},k}+\eta_{k}\epsilon_{\hat{b},k}+{\tilde{\nu }}_{k}+\epsilon_{\tilde{y}} \end{array} \end{array}$$

The measurement error mean and the covariance ($P_{\mu \mu }$) can be obtained as

(30) $$\begin{array}{cc} \; & E\left[\mu \right]=H_{k}E\left[\epsilon_{\tilde{X},k}\right]+\eta_{k}E\left[\epsilon_{\hat{b},k}\right]+E\left[{\tilde{\nu }}_{k}\right]+E\left[\epsilon_{\tilde{y}}\right]=0 \end{array}$$

(31) $$\begin{array}{cc} \; & P_{\mu \mu }=E\left[\mu \mu^{T}\right]=H_{k}\Sigma_{\tilde{X}}H_{k}^{T}+\eta_{k}\Sigma_{\hat{b}}\eta_{k}^{T}+P_{\tilde{\nu }\tilde{\nu }}+\Sigma_{\tilde{y}} \end{array}$$

where the quantization noise covariances ($\Sigma_{\tilde{X}},\Sigma_{\hat{b}}$, and $\Sigma_{\tilde{y}}$) are obtained from Equations (22)–(24) as

(32) $$\begin{array}{c} \Sigma_{\tilde{X}}=\mathrm{diag}\left(q_{x},q_{x},q_{g}\right);\;\Sigma_{\hat{b}}=q_{x};\;\mathrm{and}\;\Sigma_{\tilde{y}}=q_{y}I_{\tilde{y}} \end{array}$$

Moreover, the elements of the measurement noise covariance matrix $P_{\nu \nu }$ have explicit dependence on time and will require periodic calibration to prevent measurement degradation [27]. The covariance elements can be computed in indicial notation as

(33) $$\begin{array}{c} P_{\nu \nu ,ij}=\sigma_{m}^{2}\delta_{ij}+\Phi_{13}^{\left(i\right)}\Phi_{13}^{\left(j\right)}\left(\sigma_{b0}^{2}+\sigma_{u}^{2}\left(t_{k}-t_{0}\right)\right)+\sigma_{v}^{2}\int_{t_{k}}^{t_{k+i}}\Phi_{12}^{2}\left(t_{k+i},\tau \right)d\tau \end{array}$$

Ultimately, an optimal state estimate using the batch of measurements $\tilde{y}$, are obtained by solving the normal equations as

(34) $$\begin{array}{c} \hat{\tilde{X}}\left(k\right)={\left[H_{k}^{T}P_{\mu \mu }^{-1}H_{k}\right]}^{-1}H_{k}^{T}P_{\mu \mu }^{-1}\left(\tilde{y}-\eta_{k}\hat{b}\right) \end{array}$$

The above expression is a stochastic moving average filter, and it consumes at least three running measurements (N≥3) to provide the estimates for the single-axis position, velocity, and acceleration states at every estimation epoch. Evidently, the filter averages the measurements from two future time events, causing the filter to lag the measurement sequence by at least two measurement cycles. The minimum variance estimation with quantized states and measurements for a linear system is derived in Appendix A.

### 3.3. Note on State Estimation with Scale Factor Errors

  The readout error in Equation (26) can be modified to account for the scale factor error described in Equation (10). This modification starts with acknowledging that the measurements are contaminated by measurement noise and scale factor errors as

(35) $$\begin{array}{c} y_{k+n}=\left(1+\epsilon_{s,k+n}\right)x_{k+n}+\nu_{k+n} \end{array}$$

wherein the scale factor error is modeled as an uncorrelated zero-mean Gaussian white noise with variance $\sigma_{\epsilon_{s}}^{2}$.

As a consequence, the total readout error in Equation (26) has an additional term $\epsilon_{s,k+n}x_{k+n}$ such that

(36) $$\begin{array}{c} {\tilde{\nu }}_{k+n}=\epsilon_{s,k+n}x_{k+n}+\nu_{k+n}+\Phi_{13}^{\left(n\right)}\left(b_{k}-\hat{b}\right)+\int_{t_{k}}^{t_{k+n}}\Phi_{12}\left(t_{k+n},\tau \right)n_{v}d\tau \end{array}$$

Notice that the measurement noise is now linearly related to the displacement state. This problem can be addressed by using an *a priori* estimate of displacement state (${\hat{x}}_{a,k+n}$) from the propagation of model dynamics (Equation (15)) [35].

Now, using ${\hat{x}}_{a,k+n}$ for evaluating the scale factor error contribution, it can be shown that the additional variance that contributes to the measurement noise variance is

(37) $$\begin{array}{c} \sigma_{{\tilde{\nu }}_{k+n}}^{2}=\sigma_{\nu_{k+n}}^{2}+\sigma_{\epsilon_{s,k+n}}^{2}{\left({\hat{x}}_{a,k+n}\right)}^{2} \end{array}$$

Following a procedure similar to the one described in Section 3.2, this updated measurement noise variance can now be used to compute $P_{\tilde{\nu }\tilde{\nu }}$ in Equation (31). Note that the incorporation of *a priori* state information and corresponding *a priori* error covariance ($\tilde{Q}$) allows us to extend the least squares filter to update the state estimates as described in Appendix A.3. Additionally, since acceleration is an estimated state, its contribution to the process noise is evaluated as a tunable parameter (details in Section 3.5).

### 3.4. Multi-Oscillator Problem

The dynamic response of a harmonic oscillator depends on the oscillator’s natural frequency, damping, and the driving signal frequency. To attain a wide dynamic range for precise inertial measurements, multiple oscillators may be deployed in a multiplexed sensing network [28]. The estimates of forcing accelerations from multiple sensing nodes are appropriately fused to obtain an estimate with high confidence. If the estimates are calculated from the observations sampled by the same hardware system at the same instance, a covariance-weighted average of all the arriving estimates can be implemented to reduce noise over a wide working range of measurable accelerations using the multi-oscillator system’s response.

Assuming that the estimation errors (from least squares filter independently applied to measurements from different oscillators) are independent and unbiased (zero-mean), the acceleration estimates are fused by using a weighted average with the reciprocal variance values as weights [36]:

(38) $$\begin{array}{c} \bar{g}\left(k\right)=\frac{\sum_{i=0}^{i=N}{\hat{g}}_{i}\left(k\right).\frac{1}{P_{33}^{i}}}{\sum_{i=0}^{i=N}\frac{1}{P_{33}^{i}}} \end{array}$$

where *N* represents the number of independent estimates and $P_{33}^{i}$ is the estimated non-zero variance of ${\hat{g}}_{i}\left(k\right)$. The variance $\sigma_{\bar{g}}^{2}$ of the fused estimate is always lower or equal to the best individual estimate and is given by

(39) $$\sigma_{\bar{g}}^{2}=\sum_{i=0}^{i=N}\frac{1}{P_{33}^{i}}$$

Note that the independence of error in estimates is a loosely constructed term and difficult to fulfill, especially when the oscillators are of the same type. If the individual estimation errors are not independent, the covariance-weighted average still yields a correct estimate, but its assigned confidence is overestimated.

### 3.5. Quantized Discrete-Time Kalman Filter (QDKF)

The least squares moving average filter described in Section 3.2 uses an N+1 measurement batch to estimate the states at every epoch. In this section, a Kalman filter formulation is briefly described where the state variables are sequentially estimated by fusing predictions of the state variables from the oscillator dynamical model with noisy position measurements.

The discrete-time dynamics given in Equation (15) can be remodeled to accommodate the instantaneous forcing acceleration input as a state. However, since the forcing acceleration is not directly observed through a model, it is estimated from new measurements. The process covariance is augmented with an acceleration model uncertainty parameter α that is appropriately scaled to indicate the confidence in the evolution of $g_{k}$. After all, the acceleration input cannot be perfectly delivered to the system and is affected by a noise process that is denoted here as $w_{g,k}$. An independent periodic calibration step prevents accumulation of errors in acceleration estimates. The dynamical model therefore can be reformulated with the evolution of the modified states, $X_{k}={\left[x_{k}\;{\dot{x}}_{k}\;g_{k}\right]}^{T}$, as given by the discrete-time dynamics as

(40) $$\begin{array}{c} \underset{X_{k+1}}{\underset{︸}{\left[\begin{array}{c} x_{k+1}\\ {\dot{x}}_{k+1}\\ g_{k+1} \end{array}\right]}}=\underset{\tilde{\Phi }}{\underset{︸}{\left[\begin{array}{ccc} \Phi_{11} & \Phi_{12} & \Psi_{12}\\ \Phi_{21} & \Phi_{22} & \Psi_{22}\\ 0 & 0 & 1 \end{array}\right]}}\underset{X_{k}}{\underset{︸}{\left[\begin{array}{c} x_{k}\\ {\dot{x}}_{k}\\ g_{k} \end{array}\right]}}+\underset{\tilde{\Gamma }}{\underset{︸}{\left[\begin{array}{c} \Phi_{13}\\ \Phi_{23}\\ 0 \end{array}\right]}}\hat{b}+\underset{{\bar{w}}_{k}}{\underset{︸}{\left[\begin{array}{c} {\tilde{w}}_{k}\\ w_{g,k} \end{array}\right]}} \end{array}$$

Finally, the dynamical and the measurement models, under the influence of quantization noises, can be reformulated as

(41) $$\begin{array}{cc} X_{k+1} & ={\tilde{\Phi }}_{k}\left(X_{k}+\epsilon_{X,k}\right)+{\tilde{\Gamma }}_{k}\left(\hat{b}+\epsilon_{\hat{b},k}\right)+{\bar{w}}_{k} \end{array}$$

(42) $$\begin{array}{cc} y_{k} & =H_{k}\left(X_{k}+\epsilon_{X,k}\right)+{\tilde{\nu }}_{k}+\epsilon_{y} \end{array}$$

where $H_{k}=\left[1\;0\;0\right]$ is the observation model matrix indicating that the position of the proof mass is directly observable through measurements. The round-off errors included in the model follow the same definitions as described in the least squares estimator (Section 3.2).

With the reformulated dynamics and the observation models described in Equations (41) and (42), the round-off errors are incorporated into the Kalman filter formulation for sequential state estimation. Starting from an initial value of state and corresponding error covariance, a quantized form of Kalman filter, QDKF, is thus realized. Algorithm 1 describes the recursive operations involved in the implementation of the QDKF. The derivation of the QDKF is presented in Appendix B.
**Algorithm 1:** Quantized discrete-time Kalman filter (QDKF).  1:Initialize(43)$$\begin{array}{l} {\hat{X}}_{0}^{+}=E\left[X_{0}\right] \end{array}$$(44)$$\begin{array}{c} P_{0}^{+}=E\left[\left({\hat{X}}_{0}-X_{0}\right){\left({\hat{X}}_{0}-X_{0}\right)}^{T}\right] \end{array}$$  2:Propagate(45)$$\begin{array}{c} {\hat{X}}_{k+1}^{-}={\tilde{\Phi }}_{k}{\hat{X}}_{k}^{+}+{\tilde{\Gamma }}_{k}\hat{b} \end{array}$$(46)$$\begin{array}{c} P_{k+1}^{-}={\tilde{\Phi }}_{k}\left(P_{k}^{+}+\Sigma_{X,k}\right){\tilde{\Phi }}_{k}^{T}+{\tilde{\Gamma }}_{k}\Sigma_{\hat{b},k}{\tilde{\Gamma }}_{k}^{T}+\left[\begin{array}{cc} \tilde{Q} & 0\\ 0 & \alpha \end{array}\right] \end{array}$$  3:Update(47)$$\begin{array}{c} K_{k}=P_{k}^{-}H_{k}^{T}{\left[H_{k}P_{k}^{-}H_{k}^{T}+H_{k}\Sigma_{X,k}H_{k}^{T}+R_{k}+\Sigma_{y,k}\right]}^{-1} \end{array}$$(48)$$\begin{array}{c} {\hat{X}}_{k}^{+}={\hat{X}}_{k}^{-}+K_{k}\left(y_{k}-H_{k}{\hat{X}}_{k}^{-}\right) \end{array}$$(49)$$\begin{array}{c} P_{k}^{+}=\left[I-K_{k}H_{k}\right]P_{k}^{-} \end{array}$$

   An equivalent expression for the covariance update in Equation (49) can be written in a symmetric form, as shown below. This symmetric version is often used in software implementation as it guarantees positive semi-definiteness of $P_{k}^{+}$ in the presence of round-off errors.

(50) $$P_{k}^{+}=\left[I-K_{k}H_{k}\right]P_{k}^{-}{\left[I-K_{k}H_{k}\right]}^{T}+K_{k}\left[H_{k}\Sigma_{X,k}H_{k}^{T}+R_{k}+\Sigma_{y,k}\right]K_{k}^{T}$$

### 3.6. Quantized Square-Root Kalman Filter (QSRKF)

For onboard implementation with limited computational word-length, the standard Kalman filter algorithm is susceptible to numerical instability. Round-off errors can cause loss of positive definiteness in error covariance matrices during computation [2,37]. Square-root Kalman filters (SRKFs) mitigate this numerical degradation by computing and propagating the square roots of the error covariance matrices for both time and measurement updates. The quantized version of square-root filters, termed QSRKF here, incorporates quantization errors into the SRKF formulation, thereby improving performance as well as the filter’s numerical stability under fixed-point implementation.

In QSRKF, error covariance matrices are factored into square-root matrices computed using QR decomposition. The square roots of the initial state error, process noise, measurement noise, and quantization noise covariance matrices are calculated once using the Cholesky method. For linear time-invariant systems, these can often be predetermined and stored onboard. For the dynamical system described in the QDKF formulation (Section 3.5), Algorithm 2 presents the operations involved in QSRKF realization. The filter algorithm is described in detail in Appendix C.
**Algorithm 2:** Quantized Square-Root Kalman filter (QSRKF).  1:Initialize
(51)$$\begin{array}{cc} \; & {\hat{X}}_{0}^{+}=E\left[X_{0}\right] \end{array}$$
(52)$$\begin{array}{cc} \; & S_{0}^{+}=\sqrt{E[\left({\hat{X}}_{0}-X_{0}\right){\left({\hat{X}}_{0}-X_{0}\right)}^{T}]} \end{array}$$  2:Propagate
(53)$$\begin{array}{cc} \; & {\hat{X}}_{k+1}^{-}={\tilde{\Phi }}_{k}{\hat{X}}_{k}^{+}+{\tilde{\Gamma }}_{k}\hat{b} \end{array}$$
(54)$$\begin{array}{cc} \; & S_{k+1}^{-}=\mathrm{qr}{\left\{{\left[\Phi_{k}S_{k}^{+}\;\;|\;\;\Phi_{k}\Lambda_{x,k}\;\;\left|\;\;\Gamma_{k}\Lambda_{u,k}\;\;\right|\;\;\gamma_{k}S_{w,k}\right]}^{T}\right\}}^{T} \end{array}$$  3:Update
(55)$$\begin{array}{cc} \; & S_{zz,k}=\mathrm{qr}{\left\{{\left[H_{k}S_{k}^{-}\;\;|\;\;H_{k}\Lambda_{x,k}\;\;\left|\;\;S_{v,k}\;\;\right|\;\;\Lambda_{y,k}\right]}^{T}\right\}}^{T} \end{array}$$
(56)$$\begin{array}{cc} \; & K_{k}=S_{k}^{-}{\left(H_{k}S_{k}^{-}\right)}^{T}{\left(S_{zz,k}S_{zz,k}^{T}\right)}^{-1} \end{array}$$
(57)$$\begin{array}{cc} \; & {\hat{X}}_{k}^{+}={\hat{X}}_{k}^{-}+K_{k}\left(y_{k}-H_{k}{\hat{X}}_{k}^{-}\right) \end{array}$$
(58)$$\begin{array}{cc} \; & S_{k}^{+}=\mathrm{qr}\{{\left[\left[I-K_{k}H_{k}\right]S_{k}^{-}\;\;|\;\;K_{k}\left[H_{k}\Lambda_{x,k}\;\;\left|\;\;S_{v,k}\;\;\right|\;\;\Lambda_{y,k}\right]\right]}^{T}{\}}^{T} \end{array}$$

The following is true for the above algorithm:

- $S_{0}^{+}$ is the square-root factor of the initial estimation error covariance.
- $S_{k+1}^{-}$ and $S_{k}^{+}$ represent the prior and posterior estimation error covariance square-root factors.
- $S_{v,k}$ and $S_{w,k}$ are the square-roots of the measurement and process noise covariance matrices.
- $\Lambda_{x,k}$, $\Lambda_{y,k}$, and $\Lambda_{u,k}$ represent the matrix square-roots of the quantization error covariances for state, measurement, and output noises.
- qr{·} indicates QR decomposition operation.

## 4. Numerical Simulations

The optomechanical inertial sensor parameters and the corresponding noise processes modeled in this simulation are highlighted in Table 1. These parameters are derived from laboratory experiments and sensor benchtop prototypes described in references [28,31,38]. The contributing process noise sources, including thermal, mechanical, and cavity drifts, have been thoroughly investigated in the cited works. The oscillator parameters, process noise terms ($\sigma_{v}$, $\sigma_{u}$) driving the discrete-time state evolution, calibrated sensor bias as well as the readout noise floor [27], are set to values consistent with the experimental observations.

Noisy position measurements are simulated from true position states corrupted by a stochastic measurement noise process ($\sigma_{m}$) along with an additive quantization process ($q_{y}$). The stochastic processes ($\sigma_{v},\sigma_{u}$) driving the discrete-time state evolution, the state quantization processes ($q_{x}$), the standard deviation of the calibrated bias estimate ($\sigma_{b0}$), and its corresponding quantization process ($q_{\hat{b}}$) are also presented in Table 1.

In this simulation, sensor bias is assumed to be estimated through an independent calibration step that is briefly described in Section 2.2 and detailed in [27]. The bias estimate $\hat{b}$ is randomly drawn from $N(0,\sigma_{b0}^{2})$, and the acceleration input is simulated as a sinusoidal signal of frequency 0.01 Hz and amplitude $1\times 10^{-5}$ g. In this analysis, the state and bias values are stored with fractional word length of internal registers configured to signed 16 bits ($B_{x}=B_{b0}=16$). This means that the fractional parts of the states and input nodes are rounded off to 16 bits, with the word-length of the measurements being the only variable. The measurements are quantized to fractional lengths (FLs) of 8 to 16 bits ($B_{y}$) to emulate different ADC resolutions available on development boards. To prevent numerical underflows and to maintain precision, the covariance matrix elements are assigned higher word lengths compared with the state and measurement variables. For FPGA implementation, dedicated digital signal processing (DSP) blocks can be utilized to efficiently multiply the covariance matrix elements with the corresponding state or measurement variables. Modern FPGAs typically provide DSP blocks that support multiplication of 18-bit and 25-bit operands. To handle higher word lengths of the covariance matrix elements, multiple DSP blocks can be cascaded to perform pipelined multiply-and-accumulate operations, enabling accurate computations while maintaining high throughput.

**Figure 1:**
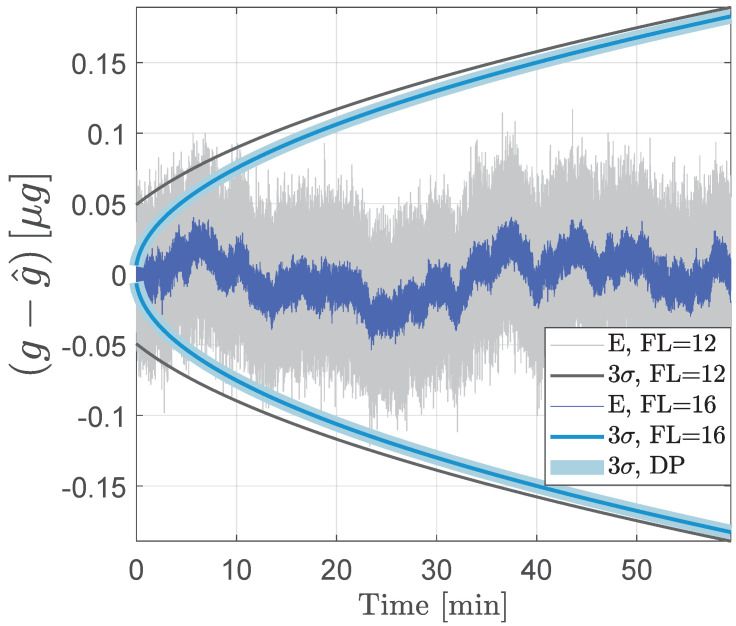
Acceleration estimation errors (E) and the corresponding 3σ bounds from the least squares-based moving average filter. The filter is run using 12 and 16-bit fractional length (FL) measurements. The 3σ bounds from double precision (DP) implementation of the least squares filter are also indicated.

Figure 1 shows the results for the errors in the estimated acceleration and the corresponding 3σ estimation error bounds from the moving average least squares method. In this figure, the errors and the 3σ bounds for the least squares implementation are compared for measurements of 12 versus 16-bit fractional length. The least squares filter structure defined in Equation (34) supplements the measurement noise covariance with the quantization error covariance that reflects the bit resolution. Hence, the simulation in Figure 1 demonstrates additional errors in the acceleration estimates due to less precise representation of measurements. The simulation also signifies the expansion of the 3σ bounds, indicative of added uncertainties in the estimated errors due to increased quantization noise in the measurements. For instance, acceleration estimates with the measurements rounded off to a fractional length of 12-bits have larger errors and covariance bounds than those of 16-bit measurements.

**Figure 2:**
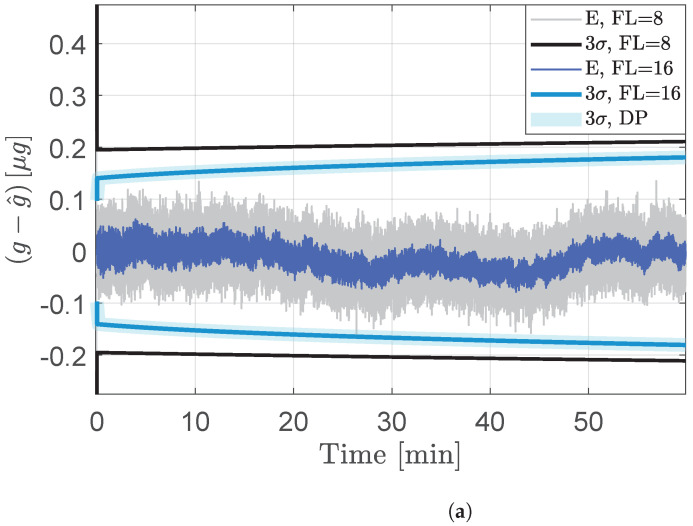

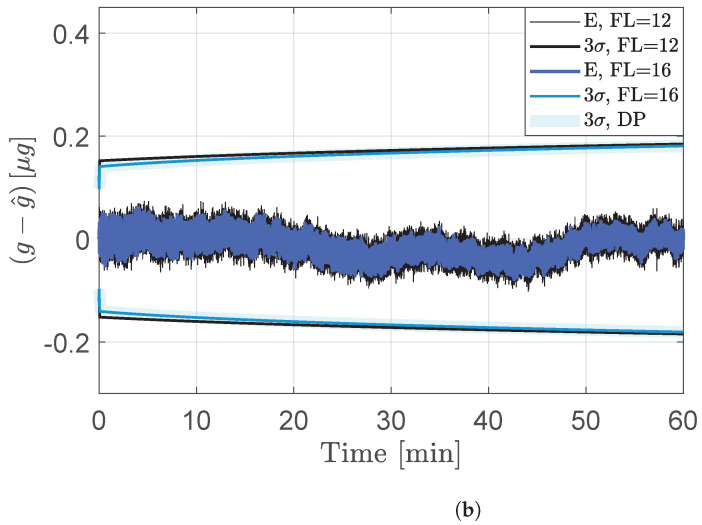
Acceleration estimation results from the quantized discrete-time Kalman filter (QDKF). The 3σ bounds from double precision (DP) simulations of the discrete-time Kalman filter without quantization errors are also shown for comparison. (**a**) Errors and corresponding 3σ bounds with quantized measurements of fractional lengths 8 and 16 bits. (**b**) Errors (E) and corresponding 3σ bounds with quantized measurements of fractional lengths of 12 and 16 bits.

Figure 2 shows the estimation error results for the Kalman filter formulation, where the process noise associated with the acceleration channel is modeled as a zero-mean process with a variance of 0.1(α=0.1). As in the least squares method, the errors and error bounds from the Kalman filter are sensitive to the precision in the measurements. This sensitivity is illustrated in Figure 2a,b, which respectively compare 8 and 12-bit measurements against measurements with 16-bit fractional resolution. The analysis suggests that as the measurement precision increases, the errors and the error bounds tend to align statistically with those of a floating-point implementation of the discrete-time Kalman filter, despite finite-precision hardware constraints. In another observation, the least squares filter has lower variance than that of the QDKF for this application. This is because the least squares filter need not account for the process noise associated with the acceleration channel.

**Figure 3:**
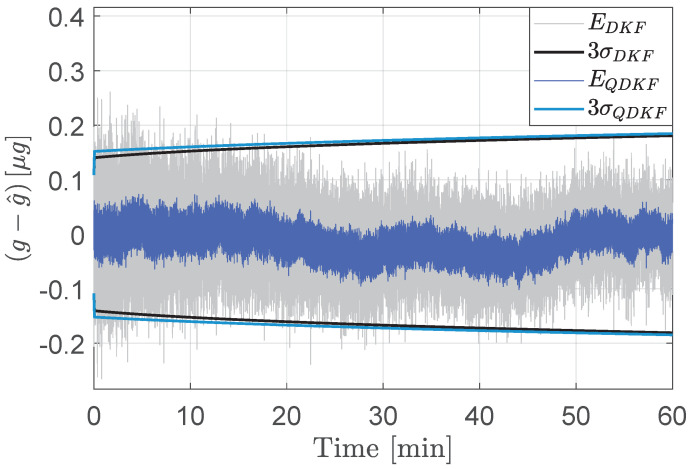
Acceleration estimation errors (E) and the corresponding 3σ bounds from the DKF and the QDKF. Measurements are quantized to fractional length of 12-bits.

Furthermore, accounting for finite word-length effects in the filter implementation not only accurately predicts the uncertainty in the estimation errors but also reduces these errors. Shown in Figure 3 is discrete-time Kalman filter (DKF) implementation without considering round-off errors in the filter design versus the quantized discrete-time Kalman filter (QDKF) proposed in this work (Algorithm 1). In this comparison, the measurements are quantized to 12-bits in both the DKF and the QDKF implementations, but the filter formulation in DKF does not incorporate quantization error covariances as the proposed QDKF algorithm does. Evidently, the QDKF filter resulted in lower error values than the DKF and also higher 3σ bounds to represent increased covariance due to quantization errors. Moreover, the DKF errors are numerically inconsistent with the corresponding error covariances, as its formulation does not account for quantization noise statistics. The same phenomenon is also evident in the least squares estimation, as observed in Figure 1. Furthermore, numerical round-off errors exacerbate the lack of observability in the bias state, resulting in growing uncertainty in acceleration estimation errors.

### 4.1. Steady-State Performance

In practice, the Kalman filter is often run for long periods of time. As k→∞ and if given input remains within reasonable magnitude, the error covariance ($P_{k}^{-}$) converges to a bounded steady-state value P. For a large *k*, $P_{k+1}^{-}=P_{k}^{-}≜P$, and Equation (46) satisfies the discrete-time algebraic Riccati equation [24]:

(59) $$\begin{array}{cc} \; & P={\tilde{\Phi }}_{k}\left[I-K_{k}H_{k}\right]P{\tilde{\Phi }}_{k}^{T}+{\tilde{\Phi }}_{k}\Sigma_{X,k}{\tilde{\Phi }}_{k}^{T}+{\tilde{\Gamma }}_{k}\Sigma_{\hat{b},k}{\tilde{\Gamma }}_{k}^{T}+\left[\begin{array}{cc} \tilde{Q} & 0\\ 0 & \alpha \end{array}\right] \end{array}$$

with $K_{k}$ given in Equation (47).

**Figure 4:**
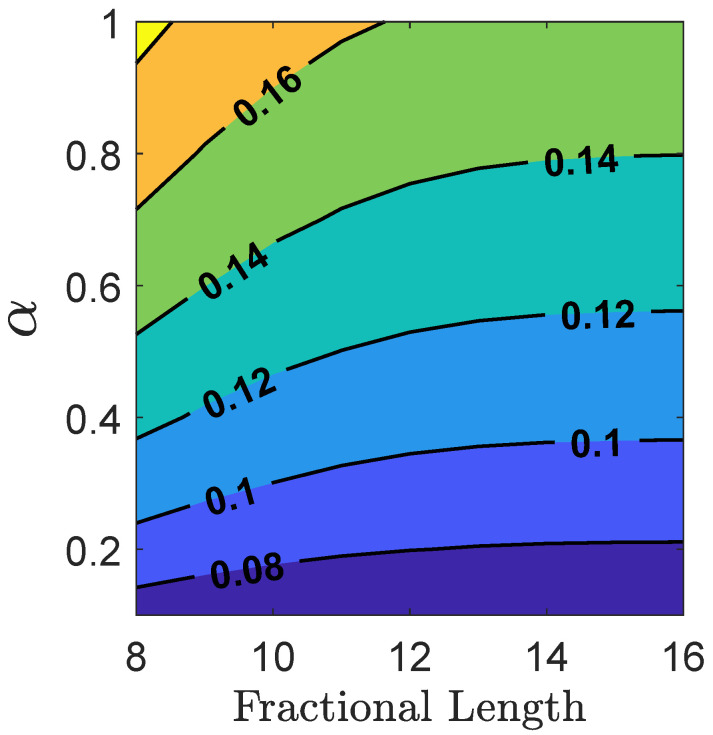
Steady-state 1σ contours for acceleration estimates. The contour lines are plotted as a function of measurement precision on the x-axis and the model uncertainty parameter for the acceleration channel, (α), on the y-axis.

Figure 4 shows the standard deviation contours for steady-state acceleration estimation errors. These contours illustrate how the steady-state error bounds change as a function of the fractional length in measurement quantization and the process noise associated with the acceleration channel. In the absence of quantization errors, the contour isolines maintain constant values for given levels of process noise. As the numerical precision of measurements increases, the contours asymptotically approach the steady-state performance achieved by floating-point implementation. The sensitivity analysis curves that graphically illustrate the impact of quantization noise on the state estimation accuracy are an important contribution of this work.

The steady-state behavior reflects the filter performance over an extended period, depicting characteristics of the estimation errors once the transient effects have diminished and the error dynamics have stabilized. The steady-state analysis offers a quantitative measure of the best possible accuracy achievable by the filter and is based on sensor parameters, process, and measurement noise characteristics. Consequently, it serves as a valuable tool in sensor design and parameter tuning. In interferometric sensing, the design and modeling of mechanical elements, signal processing, and estimation filters can be tailored for accuracy and reliability using steady-state covariance analysis.

Furthermore, in the context of quantized Kalman filtering, the Mahalanobis distance is chosen as a metric to quantify the impact of quantization noise on the consistency of estimation errors. The Mahalanobis distance (*d*) between the quantized observation ($y_{k}$) and its prediction ($H_{k}{\hat{X}}_{k}^{-})$) reflects the variance between them, scaled by the inverse of the associated covariance matrix ($P_{zz,k}^{-1}$). That is,

(60) $$\begin{array}{c} d=\sqrt{z_{k}^{T}P_{zz,k}^{-1}z_{k}} \end{array}$$

where, from Algorithm 1,

(61) $$\begin{array}{cc} z_{k} & =y_{k}-H_{k}{\hat{X}}_{k}^{-} \end{array}$$

(62) $$\begin{array}{cc} P_{zz,k} & =H_{k}P_{k}^{-}H_{k}^{T}+H_{k}\Sigma_{X,k}H_{k}^{T}+R_{k}+\Sigma_{y,k} \end{array}$$

**Figure 5:**
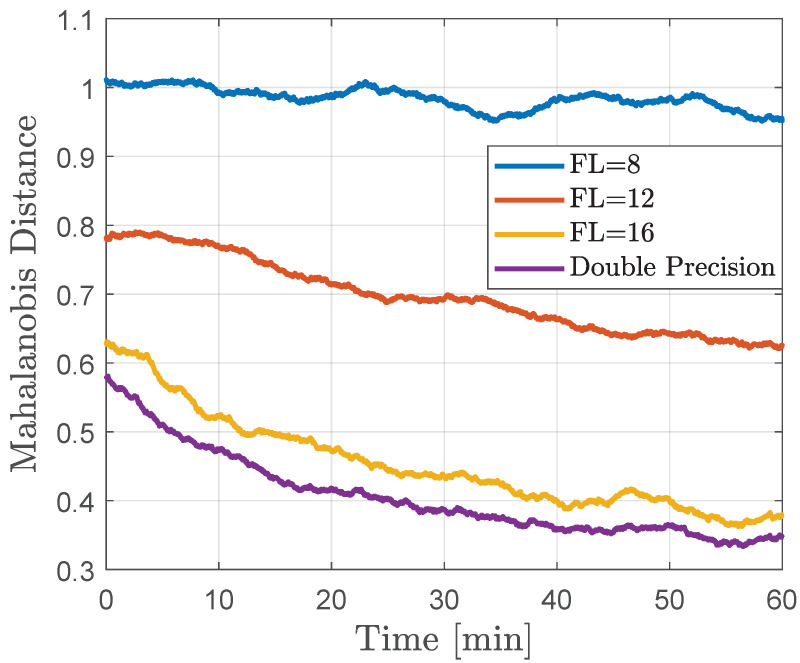
Mahalanobis distance moving average of 10,000 estimated samples.

Figure 5 illustrates the Mahalanobis distance of the estimates from the QDKF method for observations with varying fractional lengths over time. The Mahalanobis distance quantifies the dissimilarity between the estimated and true states, considering the covariance of the estimation errors. A smaller Mahalanobis distance indicates better filter performance, as the estimated states are closer to the true states. To visualize the trend, a moving average of 10,000 Mahalanobis distance samples is computed. The results demonstrate that higher quantization noise in the observations leads to greater disparities between the observed and predicted measurements. While sequential filtering helps the filter learn the quantization noise statistics over time, it cannot fully match the lower bound achieved in double-precision simulations. Notably, this metric indicates that 16-bit measurements closely track the lower bound, suggesting an optimal word length for sensor data transmissions in this application.

### 4.2. Square Root Kalman Filter Simulations

**Figure 6:**
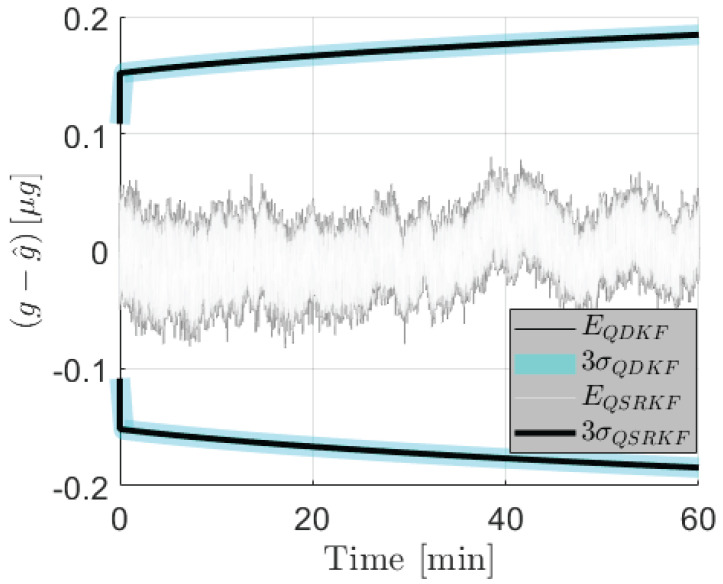
Acceleration state estimation errors (E) and the corresponding 3σ bounds from the QDKF and the QSRKF methods. Measurements are quantized to fractional length of 12-bits.

For 12-bit measurements, the variances from the quantized DKF were observed to take negative values, which is theoretically nonviable. The SRKF, by construction, avoids loss of positive definiteness of the error covariance matrices and thereby offers resistance to numerical overflows and underflows. For covariance matrices that are appropriately scaled and quantized to avoid numerical degeneracy for this application, the performance of the QSRKF proposed in Algorithm 2 is comparable to that of the QDKF (Algorithm 1). Figure 6 compares the estimation errors and the corresponding 3σ bounds from QDKF and the QSRKF filters. Although this result indicates that the errors are not significantly reduced using the QSRKF version, the loss of positive definiteness in QDKF is a weakness of the standard implementation. Therefore, the square-root filters are preferable for onboard implementation even though they are computationally burdensome.

**Figure 7:**
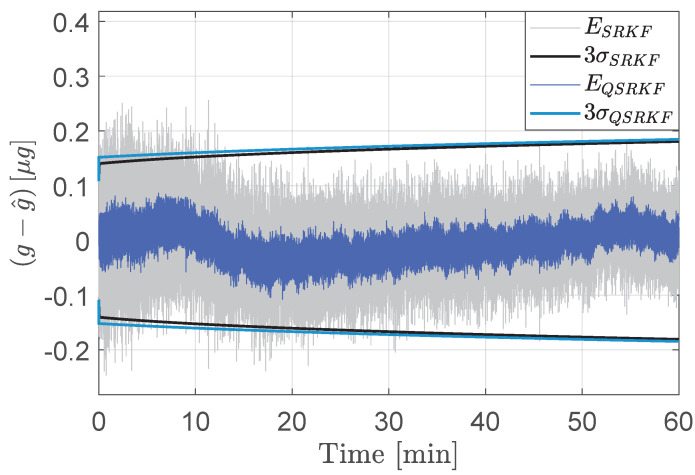
Acceleration estimation errors (E) and the corresponding 3σ bounds from the SRKF and the QSRKF. Measurements are quantized to fractional length of 12-bits.

At the same time, it is evidently important to model quantization noise sources into the standard SRKF algorithm for reliable filter performance. Figure 7 compares the performance of the square-root filters with the quantization noise modeled into the filter structure (QSRKF in Algorithm 2) versus a standard SRKF implementation that does account for the round-off error statistics. In this comparison, the measurements are quantized to 12-bits in both the QSRKF and the SRKF runs.

Table 2 presents the time-averaged Mahalanobis distance for each filter (DKF, QDKF, SRKF, and QSRKF) as a function of measurement resolutions. The results show that the quantized filters (QDKF and QSRKF) proposed in this work outperform filter implementations that do not account for round-off errors (DKF and SRKF). The quantized filters maintain a lower average Mahalanobis distance, indicating better estimation accuracy and robustness to the effects of measurement quantization. Furthermore, the average Mahalanobis distance decreases as the fractional length increases for all filters. This trend suggests that higher measurement resolutions lead to improved filter performance, as more precise measurements provide better information for state estimation. The last column in the table (DP) represents the average Mahalanobis distance for the ideal filter implementations using double-precision measurements. These values serve as a benchmark for the best achievable performance without quantization effects. The quantized filters (QDKF and QSRKF) approach the performance of the ideal filters, demonstrating their effectiveness in mitigating the impact of measurement quantization.

Numerical simulations show that the Kalman filters appear to be under-confident because of the uncertainty in the evolution of the acceleration state. The least squares filter, however, does not require handling process noise associated with the acceleration state and is therefore much more confident about the estimation errors. Moreover, the process noise of the acceleration input is well studied to have low uncertainty [28]. Therefore, a least-squares based moving average filter is considered for hardware implementation.

## 5. Architecture for FPGA Implementation

**Figure 8:**
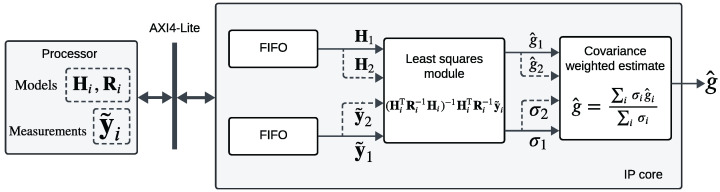
Block diagram for FPGA implementation of least squares-based covariance-weighted acceleration estimation method.

In this section, an FPGA-based embedded architecture designed for estimating input acceleration force for a dual-oscillator system is described. The estimation algorithm consumes simulated measurements from two distinct oscillator models and delivers a covariance-weighted average of the estimated accelerations as illustrated in Figure 8.

### 5.1. Implementation Overview

**Figure 9:**
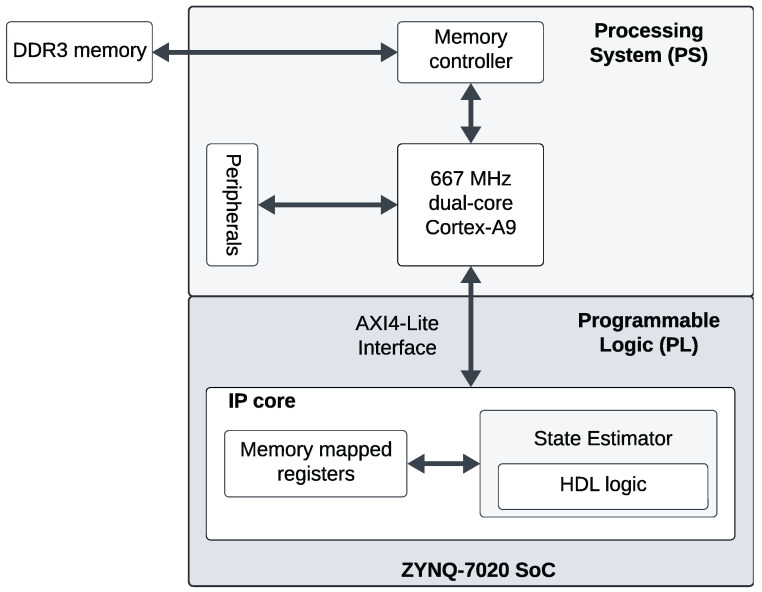
HW/SW codesign for Xilinx Zynq 7020 FPGA SoC-based acceleration state estimation: Filter-specific computations are implemented on the processing system (PS), and the filter-generic estimation algorithm is packaged as an intellectual property (IP) core and implemented on the programmable logic (PL). The 32-bit PS-PL on-chip communication bus is supported by the Advanced eXtensible Interface (AXI) protocol.

The system architecture comprises an FPGA accelerator designed to estimate the forcing accelerations from each of the oscillators within an FPGA sensor node and then to compute a covariance-weighted average of these estimates, as detailed in Section 3.4. The proposed architecture is a hardware-software co-design illustrated in Figure 8. Xilinx Zynq 7020 SoC is targeted for hardware evaluation of the proposed system. This SoC integrates a host processor, also known as the processing system (PS), featuring ARM Cortex-A9 MPCore. The PS performs the filter-specific operations that involve discrete-time state propagation and measurement model evaluation (Equations (27) and (40)). For a linear time-invariant system like the one under consideration, the state propagation from time-step $t_{k}$ to $t_{k+1}$ involves a constant state transition matrix and consequently a constant measurement model matrix (Equation (27)). Additionally, the processor manages the flow of simulated observations to be transmitted to the programmable logic (PL) via the memory-mapped register space indicated in Figure 9.

### 5.2. State Machine

**Figure 10:**
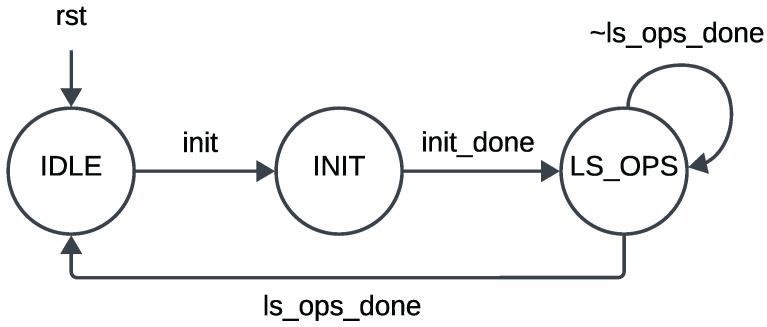
State machine for least squares operation.

The matrix operations in the intellectual property (IP) core are managed by a state machine shown in Figure 10. Initially, the IP core waits in an IDLE state, awaiting initialization by the processor. Upon receiving the initialization signal through a control register in the memory-mapped register space, the core transitions to the INIT state. Here, the processor initializes the internal memory (FIFO) of the IP core with values of the measurement model matrix augmented with the measurement noise covariance matrix. Once initialization is complete, signified by an init_done signal, the core progresses to the LS_OPS state, where least squares operations are executed. Upon completion of these operations, the core returns to the IDLE state, ready for the next batch of least squares operations, triggered by the arrival of another set of measurement model parameters relayed by the processor (for the second oscillator model in this case). This state machine governs the matrix operations within this pipelined least squares filter, with the hardware modules instantiated once and reused between different oscillator models.

In the subsequent stages of the implementation illustrated in Figure 8, the processor writes the simulated vector measurements into a data register, and the core performs the least squares estimation. Once estimates and corresponding variances from two such computations are available, the core calculates the covariance-weighted average of these estimates (Equation (38)). This result is then written by the core to a FIFO, ultimately to be read by the processor through another memory-mapped register.

### 5.3. Least Squares Module

This module implements a moving average filter that computes the normal equations using the direct expression for the least squares solution [23]. Operationally, three measurements are accumulated in a measurement vector ${\tilde{y}}_{i}$ to compute the acceleration estimate. This implies that the dimension of matrix operations is three, with the estimates having a time delay equivalent to two measurement samples.

The estimation process involves a series of fixed-point matrix operations, including matrix transpose, inversion, matrix-matrix, and matrix-vector multiplications, to arrive at the least squares solution. Matrix-matrix multiplications are performed using systolic array architecture (SAA) [39], which is a pipelined two-dimensional network of multiply and accumulate (MAC) units, effective for low-latency matrix multiply operations. Matrix-vector multiplications also utilize MAC units to operate on the time-aligned input streams of matrix and measurement vector channels. The matrix inverse is computed using direct inversion expressions for the involved 3×3 matrices. The model and measurement data required for the computation are stored in the memory buffer of the IP core.

While the least squares solution in this example is tractable because the measurement model matrices are scaled to be well-conditioned, this approach can be computationally expensive. If $H^{T}H$ is ill-conditioned, the solution can be numerically unstable [40]. In practice, Cholesky (or) QR factorization [41], singular value decomposition, and, to a reasonable extent, LU decomposition [42] are efficient and accurate methods for solving normal equations.

The least squares module is instantiated only once, and the operations are pipelined to reuse the same module for estimating accelerations from two oscillators. This ensures effective resource management on the Zynq 7020 FPGA SoC.

### 5.4. Covariance Weighted Average Module

This module reads the buffered estimates and their corresponding variances to compute a weighted average of the acceleration estimate from two oscillator units, as described in Equation (38). Fixed-point division is performed using Xilinx’s Divider Generator LogiCORE IP employing the radix-2 non-restoring integer division algorithm.

### 5.5. Implementation Results

**Figure 11:**
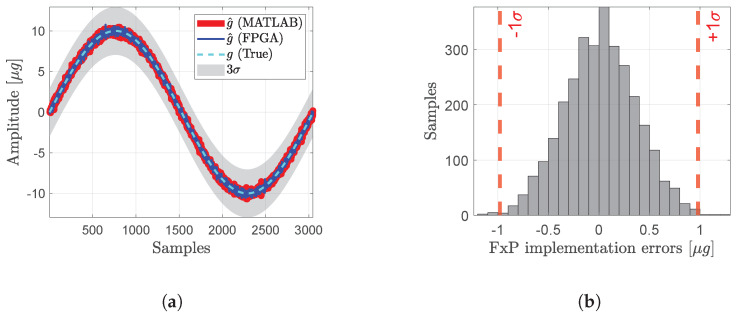
Implementation results comparing fixed-point (FxP) FPGA output with floating-point MATLAB estimates. Acceleration estimation algorithm is the covariance-weighted average from dual-oscillator system (Section 3.4). (**a**) FxP FPGA output vs. double-precision MATLAB output. (**b**) Differences between FPGA and MATLAB estimates.

The FPGA implementation of the estimation logic is validated using simulated inputs corresponding to the measurement models and the measurements themselves. To ensure accurate representation of values and avoid bit overflows and underflows, the inputs are scaled appropriately to maintain a consistent dynamic range. A MATLAB implementation of the same algorithm serves as a golden reference for comparison. The estimation results from the fixed-point FPGA implementation closely align with MATLAB’s double-precision estimates and the ground truth, as depicted in Figure 11a. Although fewer, finite-precision numerical errors as high as 10% (up to 1 μg) are observed in the FPGA output. This is illustrated in Figure 11b. These round-off errors appear to propagate through the numerous matrix operations involved, resulting in a few outlier estimates. Adaptive scaling, a higher number of fractional bits for data representation, and mixed-point (fixed and floating point) implementations are some techniques that could help reduce these errors. Additionally, solving normal equations through numerically robust methods such as Cholesky decomposition should further enhance estimation accuracy and potentially minimize the numerical errors.

### 5.6. Latency and Resource Utilization

The implementation results are shown in Table 3. This represents the implementation of state estimation logic deployed using SoC technology, where covariance-weighted acceleration estimation is implemented on an FPGA coupled to an ARM processor controlling the data flow. The hardware-software co-design required 188 digital signal processing (DSP) elements of the Zynq 7020 FPGA, running at 100 MHz. The low-latency co-design fits within 19% of the available FPGA look-up tables (LUTs), leaving the remaining resources for other potential application requirements such as sensor data processing. DSP usage can be reduced by further pipelining the operations or by strategically using LUTs for multiplications. The hardware-accelerated execution of pipelined acceleration estimation logic has an approximate latency of 3.62 μs for an estimation epoch from a dual-oscillator setup.

## 6. Conclusions

In aerospace applications, system states are filtered on onboard embedded compute elements using measurements from a sensor network. The sensors and embedded flight computing systems are resource-constrained, limiting the precision of stored or transmitted data and consequently impacting the signal-to-noise ratio of the filter output. Accurate state estimation in finite-precision embedded implementations depends on the precision of the measurements and the word lengths of the state and input variables stored on the embedded computer.

This work presents an optomechanical sensor model for estimating forcing accelerations from simulated displacement measurements of a proof mass. The state estimation algorithms are reformulated to incorporate rounding errors into classical estimator frameworks. A least squares estimator, a discrete-time Kalman filter, and a square-root Kalman filter are developed for optimal state estimation with quantized measurement, state, and input variables. Numerical simulations demonstrate that the modified filter frameworks account for finite-precision effects, ensuring proper management of errors and uncertainties in the acceleration estimates. This approach maximizes the performance of filters implemented on fixed-point hardware architectures. Steady-state performance analysis shows that the best possible accuracy achievable by the filter is tightly coupled with the numerical precision of the internal variables. Metrics such as Mahalanobis distance give concrete insights into the word-length versus performance trade-offs.

Additionally, a dual-oscillator system for estimating acceleration states from independent measurements belonging to two oscillator models is proposed for hardware implementation. A covariance-weighted average of independent acceleration estimates is realized on an FPGA-SoC using a finite-precision implementation of the least squares method. The pipelined FPGA realization with simulated model and measurements reasonably tracks a double-precision MATLAB implementation of the same least squares-based estimation. In summary, this article addresses the realization of state estimation on embedded architectures, emphasizing the importance of managing finite word-length implementation errors to design and implement high-performance, resource-efficient estimation algorithms on memory-constrained computing systems.

It is worth noting that while this work thoroughly addresses quantization effects, the potential impacts of bit overflows are neglected. Scaling digital filter realizations to prevent overflow errors remains an avenue for future investigation.

## Figures and Tables

**Table 1:** Simulated sensor model and noise parameters.

Parameter	Value	Units
**Oscillators**		
Sampling frequency ($f_{s}$)	30.5	Hz
Oscillator-1 frequency ($\omega_{1}$)	3.76	Hz
Oscillator-2 frequency ($\omega_{2}$)	8.5	Hz
Damping ratios ($\zeta_{1},\;\zeta_{2}$)	$4.386\times 10^{-6}$	
**Modeling processes**		
$\sigma_{v}$	$1\times 10^{-9}$	$m/s\sqrt{\mathrm{Hz}}$
$\sigma_{u}$	$1\times 10^{-8}$	${m/s}^{2}\sqrt{\mathrm{Hz}}$
$\sigma_{m}$	$1\times 10^{-11}$	m
$\sigma_{b0}$	$1\times 10^{-8}$	${m/s}^{2}$
**Quantization processes**		
$q_{x}$	$\sqrt{2^{-2B_{x}}/12}$	unit of corresponding state
$q_{y}$	$\sqrt{2^{-2B_{y}}/12}$	m
$q_{\hat{b}}$	$\sqrt{2^{-2B_{\hat{b}}}/12}$	m/s^2^

**Table 2:** Average Mahalanobis distance for different filters and measurement fractional lengths (FLs). DP represents the ideal filter performance with double-precision measurements.

Filter	FL = 8	FL = 10	FL = 12	FL = 16	DP
DKF	36.3042	9.2255	2.4355	0.4855	0.4018
QDKF	0.9839	0.8647	0.6923	0.4584	
SRKF	36.3031	9.1854	2.4278	0.4827	0.4016
QSRKF	0.9839	0.8647	0.6923	0.4588	

**Table 3:** Post-implementation FPGA resource utilization results.

Resource	Available	Utilization
LUTs	53,200	19%
LUTRAM	17,400	5%
Flip-Flops	106,400	14.78%
BRAM	140	0.71%
DSP	220	85.45%

## Data Availability

Dataset available on request from the authors.

## Abbreviations

The following abbreviations are used in this manuscript:

DSP	Digital Signal Processing
DoF	Degree of Freedom
FPGA	Field Programmable Gate Array
SoC	System on Chip
COTS	Commercial Off-The-Shelf
QDKF	Quantized Discrete-time Kalman Filter
QSRKF	Quantized Square-Root Kalman Filter
PS	Processing System
PL	Programmable Logic
IP	Intellectual Property
AXI	Advanced eXtensible Interface
SAA	Systolic Array Architecture
LUT	Lookup Table
BRAM	Block Random Access Memory
HDL	Hardware Description Language

## Appendix A. Least Squares Estimation with Quantized States and Measurements

In this section, the minimum variance estimation problem under the influence of quantization noise in states and measurements of a system is formulated. Following the state variable description developed by Mullis [26], Williamson and Kadiman [33], Liu and Skelton [10], and the least squares derivation by Crassidis and Junkins [35], the round-off errors are incorporated for minimum variance state estimation.

### Appendix A.1. The Minimum Variance State Estimation Problem

Consider a discrete-time dynamical system:

(A1) $$\begin{array}{c} x_{i}=\Phi \left(t_{i},t_{k}\right)x_{k} \end{array}$$

and the observational system given by:

(A2) $$\begin{array}{c} z_{i}={\tilde{H}}_{i}x_{i}+\nu_{i} \end{array}$$

where $E\{\nu_{i}\}=0$∀i and $E\left\{\nu_{i}\nu_{j}^{T}\right\}=R_{ij}$.

The above systems assume ideal i.e., infinite precision in states and measurements. However, in finite word length digital compute elements, the system states $x_{i}$ and the measurements $z_{i}$ will be quantized. A fixed-point finite word-length realization of the ideal systems in which quantization is implemented after accumulation of products is described by:

(A3) $$\begin{array}{c} \begin{array}{cc} \; & x_{i}=\Phi \left(t_{i},t_{k}\right)Q\left[x_{k}\right]\\ \; & z_{i}={\tilde{H}}_{i}Q\left[x_{i}\right]+\nu_{i} \end{array} \end{array}$$

where Q[.] represents the quantization operator and $Q\left[x_{i}\right],Q\left[z_{i}\right]$ represent the quantized values of the system state and observation vectors, $x_{i},z_{i}$, respectively.

Assuming the additive property of round-off errors, the quantization process affects the states and measurements as:

(A4) $$\begin{array}{c} \begin{array}{c} Q\left[x_{i}\right]=x_{i}+\epsilon_{x,i}\\ Q\left[z_{i}\right]=z_{i}+\epsilon_{z,i} \end{array} \end{array}$$

The round-off errors, $\epsilon_{x,i}\;\mathrm{and}\;\epsilon_{z,i}$, can be modeled as zero-mean, uncorrelated white noise sequences [34] with the error statistics described as:

(A5) $$\begin{array}{c} \begin{array}{cc} \; & E\left\{\epsilon_{x,i}\right\}=0\;\forall \;i\;\mathrm{and}\;E\left\{\epsilon_{x,i}\epsilon_{x,j}^{T}\right\}=qI_{x};\;q=\frac{2^{-2B_{x}}}{12}\\ \; & E\left\{\epsilon_{z,i}\right\}=0\;\forall \;i\;\mathrm{and}\;E\left\{\epsilon_{z,i}\epsilon_{z,j}^{T}\right\}=qI_{z};\;q=\frac{2^{-2B_{z}}}{12}\\ \; & E\{\epsilon_{x,i}\epsilon_{z,i}^{T}\}=0 \end{array} \end{array}$$

The components $Q[x_{i}]$ and $Q[z_{i}]$ are assumed to be quantized to have $B_{x}$-bit and $B_{z}$-bit fractional representations, respectively. This assumption arises from the need to quantize the states to a desired finite-length ($B_{x}$ bits) on the compute element and store them for subsequent calculations, while the measurements are quantized by an A/D converter. The model coefficients also have finite length but this implementation assumes rounding-off of the product of model coefficients and the state variables. For example, if ${\tilde{H}}_{i}$ is quantized to $B_{c}$ bits, the product ${\tilde{H}}_{i}Q\left[x_{i}\right]$ results in a $B_{x}+B_{c}$ bit fraction, and the result is quantized to $B_{x}$ bits for subsequent operations. In essence, this approach does not directly optimize on the model coefficient errors but can only provide evaluation of the filter performance with respect to coefficient errors [10].

Substituting Equation (A4) into Equation (A3) and incorporating quantization in measurements to obtain the quantized state and observation systems as:

(A6) $$\begin{array}{c} \begin{array}{cc} \; & x_{i}=\Phi \left(t_{i},t_{k}\right)x_{k}+\Phi \left(t_{i},t_{k}\right)\epsilon_{x,k}\\ \; & z_{i}={\tilde{H}}_{i}x_{i}+{\tilde{H}}_{i}\epsilon_{x,i}+\nu_{i}+\epsilon_{z,i} \end{array} \end{array}$$

Here, processing a collection of measurements to estimate $x_{k}$ through the use of state transition matrix amounts to:

(A7) $$\begin{array}{c} z=Hx_{k}+H\epsilon_{x}+\nu +\epsilon_{z} \end{array}$$

where

$$\begin{array}{c} z=\left[\begin{array}{c} z_{1}\\ z_{2}\\ \vdots \end{array}\right],\;H=\left[\begin{array}{c} {\tilde{H}}_{1}\Phi \left(t_{1},t_{k}\right)\\ {\tilde{H}}_{2}\Phi \left(t_{2},t_{k}\right)\\ \vdots \end{array}\right],\;\epsilon_{x}=\left[\begin{array}{c} \epsilon_{x,1}\\ \epsilon_{x,2}\\ \vdots \end{array}\right],\;\nu =\left[\begin{array}{c} \nu_{1}\\ \nu_{2}\\ \vdots \end{array}\right],\;\epsilon_{z}=\left[\begin{array}{c} \epsilon_{z,1}\\ \epsilon_{z,2}\\ \vdots \end{array}\right] \end{array}$$

A shorthand representation of Equation (A7) is:

(A8) $$\begin{array}{c} z=Hx_{k}+\mu \end{array}$$

where all the noise-related terms are combined into a new variable μ such that

(A9) $$\mu =H\epsilon_{x}+\nu +\epsilon_{z}$$

Before deriving the estimator, let’s define the statistics of measurement and quantization noises in Equation (A9). For the collection of measurements, the first and the second central moments are described as:

(A10) $$\begin{array}{c} E\left\{\nu \right\}={\left[0\;0\;\ldots \;\right]}^{T}\;\mathrm{and}\;E\left\{\nu \nu^{T}\right\}=P_{\nu \nu } \end{array}$$

The central moments of the quantization noise in states and the measurements are described in Equation (A5) and noted here as:

(A11) $$\begin{array}{c} E\left\{\epsilon_{x}\right\}={\left[0\;0\;\ldots \;\right]}^{T}\;\mathrm{and}\;E\left\{\epsilon_{x}\epsilon_{x}^{T}\right\}=\Sigma_{x} \end{array}$$

(A12) $$\begin{array}{c} E\left\{\epsilon_{z}\right\}={\left[0\;0\;\ldots \;\right]}^{T}\;\mathrm{and}\;E\left\{\epsilon_{z}\epsilon_{z}^{T}\right\}=\Sigma_{z} \end{array}$$

Finally, the mean and covariance of the combined noise model μ is:

(A13) $$\begin{array}{c} \begin{array}{cc} E\{\mu \} & =E\{H\epsilon_{x}+\nu +\epsilon_{z}\}\\ \; & =HE\left\{\epsilon_{x}\right\}+E\left\{\nu \right\}+E\left\{\epsilon_{z}\right\}\\ \; & =H.0+0+0=0 \end{array} \end{array}$$

(A14) $$\begin{array}{c} \begin{array}{cc} P_{\mu \mu } & =E\{\mu \mu^{T}\}\\ \; & =E\{\left[H\epsilon_{x}+\nu +\epsilon_{z}\right]{\left[H\epsilon_{x}+\nu +\epsilon_{z}\right]}^{T}\} \end{array} \end{array}$$

wherein identifying that the errors are mutually uncorrelated and the definitions of individual covariances follows that

(A15) $$\begin{array}{c} \begin{array}{cc} P_{\mu \mu }\; & =HE\left\{\epsilon_{x}\epsilon_{x}^{T}\right\}H^{T}+E\left\{\nu \nu^{T}\right\}+E\left\{\epsilon_{z}\epsilon_{z}^{T}\right\}\\ \; & =H\Sigma_{x}H^{T}+P_{\nu \nu }+\Sigma_{z} \end{array} \end{array}$$

### Appendix A.2. Linear, Unbiased, Minimum Variance Estimation

The objective of the estimator is to seek a linear, unbiased, minimum variance estimate, ${\hat{x}}_{k}$ of the state $x_{k}$.

**Linear**. To begin with, the desired estimate, ${\hat{x}}_{k}$, is a linear combination of measurements. That is

(A16) $$\begin{array}{c} {\hat{x}}_{k}=Mz+n \end{array}$$

where, for *n* number of states and *m* number of measurements, an optimal choice of $M\in R^{n\times m}$ and $n\in R^{n\times 1}$ is to be determined.

Additionally, for a perfect set of measurements i.e., $\nu_{i}=0\;\;\forall \;\;i$, and a perfect measurement model z=Hx(k), the measurement system in Equation (A7) should result in the true state x(k) such that

(A17) $$\begin{array}{c} \begin{array}{c} {\hat{x}}_{k}=x_{k}=Mz+n\\ x_{k}=MHx_{k}+n \end{array} \end{array}$$

**Unbiased**. Next, for an unbiased estimate, the expected value of the estimated state should be the true state. This, combined with the linear model assumption in Equation (A16), gives:

(A18) $$\begin{array}{cc} \; & E\left\{{\hat{x}}_{k}\right\}=x_{k} \end{array}$$

(A19) $$\begin{array}{cc} \; & E\left\{Mz+n\right\}=x_{k} \end{array}$$

Then, from the assumed measurement model, it follows that

(A20) $$\begin{array}{c} E\left\{M\left[Hx_{k}+\mu \right]+n\right\}=E\left\{MHx_{k}+M\mu +n\right\}=x_{k} \end{array}$$

Since, the matrices M, H and the vector n are deterministic and the noise is a zero-mean process (Equation (A13)), it follows that

(A21) $$\begin{array}{c} \begin{array}{cc} \; & MHE\left\{x_{k}\right\}+ME\left\{\mu \right\}+n=x_{k}\\ \; & MHx_{k}+n=x_{k} \end{array} \end{array}$$

where M and n satisfy the constraints:

(A22) $$\begin{array}{c} MH=I_{n}\;\mathrm{and}\;n=0 \end{array}$$

**Minimum Variance**. As a final condition, an optimal minimum variance estimator is the one that has the smallest variance of all possible estimators. The objective is to minimize the state covariance to find an optimal choice of M.

If $e_{x}k$ defines the state estimation error such as $e_{x}k=x_{k}-{\hat{x}}_{k}$, the error mean and covariances are identified as:

(A23) $$\begin{array}{cc} E\{e_{x,k}\} & =E\left\{x_{k}-{\hat{x}}_{k}\right\}=E\left\{x_{k}\right\}-E\left\{{\hat{x}}_{k}\right\}=0 \end{array}$$

(A24) $$\begin{array}{cc} P_{\mathrm{xx},k} & =E\{\left[x_{k}-{\hat{x}}_{k}\right]{\left[x_{k}-{\hat{x}}_{k}\right]}^{T}\} \end{array}$$

Using the quantized observational system (Equation (A7)) and the linear estimation model (Equation (A16)) in the above expression for covariance gives:

(A25) $$\begin{array}{cc} P_{\mathrm{xx},k} & =E\{\left[x_{k}-\mathrm{Mz}\right]{\left[x_{k}-\mathrm{Mz}\right]}^{T}\}\\ \; & =E\{\left[x_{k}-M\left(Hx_{k}+\mu \right)\right]{\left[x_{k}-M\left(Hx_{k}+\mu \right)\right]}^{T}\}\\ \; & =MP_{\mu \mu }M^{T} \end{array}$$

where the constraint $\mathrm{MH}=I_{n}$ is used to eliminate $x_{k}$’s in the second step while the fact that M is deterministic is used in the second last step.

#### Constrained Optimization

The goal is to minimize the covariance matrix $P_{\mathrm{xx},k}$ to determine M while respecting the constraint on it. The covariance to minimize becomes:

(A26) $$\begin{array}{c} P_{\mathrm{xx},k}=MP_{\mu \mu }M^{T}+\Lambda^{T}{\left[I_{n}-\mathrm{MH}\right]}^{T}+\left[I_{n}-\mathrm{MH}\right]\Lambda \end{array}$$

wherein the constraint on M is accounted using a matrix of Lagrange multipliers, Λ, and the fact that $P_{\mathrm{xx},k}$ is symmetric is respected by adding the transpose of the constraint term.

Setting the first variation of $P_{\mathrm{xx},k}$ in the above equation to zero i.e., $\delta P_{\mathrm{xx},k}=0$, gives two simultaneous conditions:

(A27) $$\begin{array}{cc} MP_{\mu \mu }-\Lambda^{T}H^{T} & =0\;\mathrm{and} \end{array}$$

(A28) $$\begin{array}{cc} I_{n}-\mathrm{MH} & =0 \end{array}$$

Here $P_{\mu \mu }$ is a positive definite matrix, therefore from the above equation M is determined as:

(A29) $$\begin{array}{c} M=\Lambda^{T}H^{T}P_{\mu \mu }^{-1} \end{array}$$

substituting M in Equation (A28) gives an expression for the Lagrange multiplier matrix as:

(A30) $$\begin{array}{c} \Lambda^{T}={\left[H^{T}P_{\mu \mu }^{-1}H\right]}^{-1} \end{array}$$

Using the Equation (A30) in Equation (A29), the optimal choice of M that minimizes the covariance $P_{\mathrm{xx}}\left(k\right)$, is obtained as:

(A31) $$\begin{array}{c} M={\left[H^{T}P_{\mu \mu }^{-1}H\right]}^{-1}H^{T}P_{\mu \mu }^{-1} \end{array}$$

Finally, the unbiased estimate $\hat{x}\left(k\right)$ that is linear in measurements z is given as:

(A32) $$\begin{array}{cc} {\hat{x}}_{k} & =Mz\\ {\hat{x}}_{k} & ={\left[H^{T}P_{\mu \mu }^{-1}H\right]}^{-1}H^{T}P_{\mu \mu }^{-1}z \end{array}$$

Also, the covariance matrix can be derived by substituting M in $P_{\mathrm{xx}}\left(k\right)=MP_{\mu \mu }M^{T}$ as:

(A33) $$\begin{array}{c} P_{\mathrm{xx},k}={\left[H^{T}P_{\mu \mu }^{-1}H\right]}^{-1} \end{array}$$

This derivation is similar to the classical minimum variance estimator with measurement errors. However, from re-deriving the estimator in the presence of round-off errors in states and measurements, the unbiased estimates and estimation covariance are observed to be interestingly impacted. Firstly, while the least squares filter structure appears to remain unaffected by round-off errors, it necessitates careful attention to the elements involved in the structure. Notably, the covariance matrix is now influenced by the state quantization noise that is propagated through the system dynamics. The measurement quantization errors further expand the covariance matrix. The resulting least squares filter serves as an optimal estimator of state nodes quantized after each accumulation epoch, derived from quantized measurements. The filter expressions are summarized in Table A1.

**Table A1:** Quantized minimum variance estimator.

State estimate	${\hat{x}}_{k}$ = ${\left[H^{T}P_{\mu \mu }^{-1}H\right]}^{-1}H^{T}P_{\mu \mu }^{-1}z$
State error covariance	$P_{\mathrm{xx},k}$ = ${\left[H^{T}P_{\mu \mu }^{-1}H\right]}^{-1}$
Measurement error covariance	$P_{\mu \mu }$ = $H\Sigma_{x}H^{T}+P_{\nu \nu }+\Sigma_{z}$

### Appendix A.3. Estimation with a Priori Information

In the least squares formulation of Equation (A32), the *a priori* knowledge of the model dynamics can be rigorously incorporated to obtain an updated state estimate based on the recently seen information. The same formulation as that of the least squares filter can be extended to incorporate prior information about the state [35]. Thereby, a maximum *a posteriori* estimate under the presence of *a priori* state information ${\hat{x}}_{a}$ and an *a priori* error covariance matrix $Q_{a}$ can be written as:

(A34) $$\begin{array}{c} \hat{x}\left(k\right)={\left[H^{T}P_{\mu \mu }^{-1}H+Q_{a}^{-1}\right]}^{-1}\left[H^{T}P_{\mu \mu }^{-1}z+Q_{a}^{-1}{\hat{x}}_{a,k}\right] \end{array}$$

(A35) $$\begin{array}{c} P_{\mathrm{xx},k}={\left[H^{T}P_{\mu \mu }^{-1}H+Q_{a}^{-1}\right]}^{-1} \end{array}$$

## Appendix B. Quantized Discrete-Time Kalman Filter (QDKF)

Practical implementation of the Kalman filter often encounters numerous challenges. One frequent issue is due to filter divergence when actual estimation errors statistically deviate from computed estimation errors [43]. This discrepancy can lead to estimation errors exceeding the confidence intervals defined by the computed error covariance, which may approach infinity. Consequently, the filter fails to reach a steady state, especially as the measurement interval tends to infinity. Such anomalies may stem from errors in the system model upon which the filter relies or inaccuracies in modeling the error statistics [44].

This work introduces a theoretical framework for modeling errors in Kalman filter implementation on finite-precision hardware. The derivation involves incorporating quantization noises into the filter design. It will be demonstrated that integrating quantization errors into the model renders the resulting filter sensitive to the embedded architecture on which it is implemented. Moreover, embedding such errors enhances the filter’s ability to recover from otherwise unmodeled errors.

### Appendix B.1. Kalman Filter Derivation

Following the assumptions on quantization noise properties that are put forth in the least squares formulation (Appendix A.1), this derivation considers a linear, discrete-time dynamical system defined by the difference equation as:

(A36) $$\begin{array}{c} x_{k+1}=\Phi_{k}x_{k}+\Gamma_{k}u_{k}+\gamma_{k}w_{k} \end{array}$$

where, at time *k*, $x_{k}$ is the system state, $u_{k}$ is the control input, $w_{k}$ is additive process noise, $\Phi_{k}$ is the state transition matrix, $\Gamma_{k}$ is the input transition matrix, and $\gamma_{k}$ is a deterministic matrix that maps process noise into the state dynamics.

Further, the measurements are linearly related to the states and are available in discrete-time form as:

(A37) $$\begin{array}{c} y_{k}=H_{k}x_{k}+v_{k} \end{array}$$

where $y_{k}$ is the measurement obtained at time *k*, $H_{k}$ is the observation matrix, and $v_{k}$ represents the additive noise that corrupts the measurements. Note that $w_{k}$ and $v_{k}$ are assumed to be uncorrelated zero-mean Gaussian white-noise processes with variances defined as:

(A38) $$\begin{array}{c} \begin{array}{c} E\left\{w_{k}w_{l}^{T}\right\}=Q_{k}\delta_{kl}\\ E\left\{v_{k}v_{l}^{T}\right\}=R_{k}\delta_{kl} \end{array}\;\delta_{kl}=\left\{\begin{array}{cc} 1, & \mathrm{if}\;k=l.\\ 0, & \mathrm{if}\;k\neq l. \end{array}\right. \end{array}$$

Furthermore, it is supposed that the initial system state has a known mean and covariance, ${\hat{x}}_{0}$ and $P_{0}$ respectively defined as:

(A39) $$\begin{array}{c} {\hat{x}}_{0}=E\left[x_{0}\right]\;\mathrm{and}\;P_{0}=E\left[\left({\hat{x}}_{0}-x_{0}\right){\left({\hat{x}}_{0}-x_{0}\right)}^{T}\right] \end{array}$$

#### Appendix B.1.1. Quantization Assumptions

In the finite-precision implementation of the Kalman filter, the round-off errors are to be modeled in the filter derivation. Assuming that quantization (Q[.]) is implemented at state nodes after double-length accumulation, and that measurements from A/D conversion as well as input resulting from D/A conversion are independently quantized, the additional quantization errors effect the states, measurements and inputs as follows:

(A40) $$\begin{array}{c} Q\left[x_{k}\right]=x_{k}+\epsilon_{x,k} \end{array}$$

(A41) $$\begin{array}{c} Q\left[y_{k}\right]=y_{k}+\epsilon_{y,k} \end{array}$$

(A42) $$\begin{array}{c} Q\left[u_{k}\right]=u_{k}+\epsilon_{u,k} \end{array}$$

Following the description in Equation (A5), the round-off errors $\epsilon_{x,k},\;\epsilon_{y,k},\;\mathrm{and}\;\epsilon_{u,k}$, can be modeled as zero-mean, uncorrelated white noise process with the error statistics defined as:

(A43) $$\begin{array}{c} \begin{array}{cc} \; & E\left\{\epsilon_{x,k}\right\}=0\;\forall \;i\;\mathrm{and}\;\Sigma_{x,k}=E\left\{\epsilon_{x,k}\epsilon_{x,l}^{T}\right\}=qI_{x};\;q=\frac{2^{-2B_{x}}}{12}\\ \; & E\left\{\epsilon_{y,k}\right\}=0\;\forall \;i\;\mathrm{and}\;\Sigma_{y,k}=E\left\{\epsilon_{y,k}\epsilon_{y,l}^{T}\right\}=qI_{y};\;q=\frac{2^{-2B_{y}}}{12}\\ \; & E\left\{\epsilon_{u,k}\right\}=0\;\forall \;i\;\mathrm{and}\;\Sigma_{u,k}=E\left\{\epsilon_{u,k}\epsilon_{u,l}^{T}\right\}=qI_{u};\;q=\frac{2^{-2B_{u}}}{12} \end{array} \end{array}$$

These additional errors for finite-precision implementation can be modeled into the filter description in Equations (A36) and (A37) as:

(A44) $$\begin{array}{cc} x_{k+1} & =\Phi_{k}\left(x_{k}+\epsilon_{x,k}\right)+\Gamma_{k}\left(u_{k}+\epsilon_{u,k}\right)+\gamma_{k}w_{k} \end{array}$$

(A45) $$\begin{array}{cc} y_{k} & =H_{k}\left(x_{k}+\epsilon_{x,k}\right)+v_{k}+\epsilon_{y,k} \end{array}$$

#### Appendix B.1.2. Derivation

Given the above assumptions, the objective is to determine an optimal estimate of the state at ${\left(k+1\right)}^{\mathrm{th}}$ instance i.e., ${\hat{x}}_{k+1}$ based upon a set of k+1 sets of measurements and the current state estimate at *k*. The Kalman filter comprises of propagation and update stages for states and covariances. The propagation stage predicts *a priori* mean and error covariance of the state while the update stage uses new measurements to update the prediction to yield *a posteriori* mean and error covariance.

The propagation of the state is attained by taking an expected value of the difference equation in Equation (A44). The mean and covariances are obtained as follows:

(A46) $$\begin{array}{cc} {\hat{x}}_{k+1} & =E[\Phi_{k}x_{k}+\Phi_{k}\epsilon_{x,k}+\Gamma_{k}u_{k}+\Gamma_{k}\epsilon_{u,k}+\gamma_{k}w_{k}]\\ \; & =\Phi_{k}E\left[x_{k}\right]+\Gamma_{k}E\left[u_{k}\right]\\ {\hat{x}}_{k+1} & =\Phi_{k}{\hat{x}}_{k}+\Gamma_{k}u_{k} \end{array}$$

In arriving at this result, deterministic nature of the transition matrices and the input vector as well as zero-mean properties of the process and quantization noises is utilized. The covariance propagation stems from the state estimation errors at discrete instances k+1 and *k* determined as:

(A47) $$\begin{array}{cc} e_{x,k+1} & =x_{k+1}-{\hat{x}}_{k+1}\\ \; & =\left[\Phi_{k}\left(x_{k}+\epsilon_{x,k}\right)+\Gamma_{k}\left(u_{k}+\epsilon_{u,k}\right)+\gamma_{k}w_{k}\right]-\left[\Phi_{k}{\hat{x}}_{k}+\Gamma_{k}u_{k}\right]\\ \; & =\Phi_{k}\left(e_{x,k}+\epsilon_{x,k}\right)+\Gamma_{k}\epsilon_{u,k}+\gamma_{k}w_{k} \end{array}$$

where $e_{x,k}=x_{k}-{\hat{x}}_{k}$.

Now the estimation error covariance at ${\left(k+1\right)}^{\mathrm{th}}$ instance, $P_{k+1}$, is determined using the above error propagation equation as:

(A48) $$\begin{array}{cc} P_{k+1} & =E\{e_{x,k+1}e_{x,k+1}^{T}\}\\ \; & =E\{\left[\Phi_{k}\left(e_{x,k}+\epsilon_{x,k}\right)+\Gamma_{k}\epsilon_{u,k}+\gamma_{k}w_{k}\right]+{\left[\Phi_{k}\left(e_{x,k}+\epsilon_{x,k}\right)+\Gamma_{k}\epsilon_{u,k}+\gamma_{k}w_{k}\right]}^{T}\}\\ \; & =\Phi_{k}E\left\{\left(e_{x,k}+\epsilon_{x,k}\right){\left(e_{x,k}+\epsilon_{x,k}\right)}^{T}\right\}\Phi_{k}^{T}+\Gamma_{k}E\left\{\epsilon_{u,k}\epsilon_{u,k}^{T}\right\}\Gamma_{k}^{T}+\gamma_{k}E\left\{w_{k}w_{k}^{T}\right\}\gamma_{k}^{T}\\ P_{k+1} & =\Phi_{k}\left(P_{k}+\Sigma_{x,k}\right)\Phi_{k}^{T}+\Gamma_{k}\Sigma_{u,k}\Gamma_{k}^{T}+\gamma_{k}Q_{k}\gamma_{k}^{T} \end{array}$$

wherein the above expression is obtained from the information that $\Phi_{k},\Gamma_{k}$ and $\gamma_{k}$ are deterministic., the noise terms are uncorrelated as defined in the Equations (A38) and (A43).

Completing the propagation step gives prior mean and error covariance of the state which from now on be called as ${\hat{x}}_{k}^{-}$ and $P_{k}^{-}$, respectively. After the propagation step, the new measurement information updates the estimated state and the confidence in that estimate. In the step it is desired to update the prior estimate of the state and error covariance $\left\{{\hat{x}}_{k}^{-},\;P_{k}^{-}\right\}$ to produce *a posteriori* mean and covariance, $\left\{{\hat{x}}_{k}^{+},\;P_{k}^{+}\right\}$. A linear update equation for the new estimate combines the prior estimate with measurement data and can be expressed as:

(A49) $$\begin{array}{cc} {\hat{x}}_{k}^{+} & ={\hat{x}}_{k}^{-}+K_{k}\left(y_{k}-H_{k}{\hat{x}}_{k}^{-}\right) \end{array}$$

(A50) $$\begin{array}{cc} {\hat{x}}_{k}^{+} & =\left[I-K_{k}H_{k}\right]{\hat{x}}_{k}^{-}+K_{k}y_{k} \end{array}$$

where $K_{k}$ is the Kalman gain which multiplies innovation and adds it to the previous best estimate of the state. The difference between the actual measurement and its prediction i.e., $\left(y_{k}-H_{k}{\hat{x}}_{k}^{-}\right)$, is known as the innovation, $z_{k}$. The expression for the covariance of the innovation, $P_{zz,k}$, will be derived shortly.

From the update equation in Equation (A45) and the measurement model described in Equation (A49), the posterior estimation error can be computed as:

(A51) $$\begin{array}{cc} e_{x,k}^{+} & =x_{k}-{\hat{x}}_{k}^{+}\\ \; & =x_{k}-\left(\left[I-K_{k}H_{k}\right]{\hat{x}}_{k}^{-}+K_{k}y_{k}\right)\\ \; & =x_{k}-\left(\left[I-K_{k}H_{k}\right]{\hat{x}}_{k}^{-}+K_{k}\left[H_{k}\left(x_{k}+\epsilon_{x,k}\right)+v_{k}+\epsilon_{y,k}\right]\right)\\ \; & =\left[I-K_{k}H_{k}\right]\left(x_{k}-{\hat{x}}_{k}^{-}\right)-K_{k}\left[H_{k}\epsilon_{x,k}+v_{k}+\epsilon_{y,k}\right]\\ e_{x,k}^{+} & =\left[I-K_{k}H_{k}\right]e_{x,k}^{-}-K_{k}\left[H_{k}\epsilon_{x,k}+v_{k}+\epsilon_{y,k}\right] \end{array}$$

Now, the posterior estimation error covariance from the posterior state estimation error is obtained as:

(A52) $$\begin{array}{cc} P_{k}^{+} & =E\{e_{x,k}^{+}{e_{x,k}^{+}}^{T}\}\\ \; & =E\{\left(\left[I-K_{k}H_{k}\right]e_{x,k}^{-}-K_{k}\left[H_{k}\epsilon_{x,k}+v_{k}+\epsilon_{y,k}\right]\right)\\ \; & \;{\left(\left[I-K_{k}H_{k}\right]e_{x,k}^{-}-K_{k}\left[H_{k}\epsilon_{x,k}+v_{k}+\epsilon_{y,k}\right]\right)}^{T}\}\\ \; & =\left[I-K_{k}H_{k}\right]E\left\{e_{x,k}^{-}{e_{x,k}^{-}}^{T}\right\}{\left[I-K_{k}H_{k}\right]}^{T}+\\ \; & \;K_{k}\left[H_{k}E\left\{\epsilon_{x,k}\epsilon_{x,k}^{T}\right\}H_{k}^{T}+E\left\{v_{k}v_{k}^{T}\right\}+E\left\{\epsilon_{y,k}\epsilon_{y,k}^{T}\right\}\right]K_{k}^{T}\\ P_{k}^{+} & =\left[I-K_{k}H_{k}\right]P_{k}^{-}{\left[I-K_{k}H_{k}\right]}^{T}+K_{k}\left[H_{k}\Sigma_{x,k}H_{k}^{T}+R_{k}+\Sigma_{y,k}\right]K_{k}^{T} \end{array}$$

Equation (A52) is the posterior error covariance update that is derived assuming the Kalman gain $K_{k}$ to be deterministic.

In order to determine $K_{k}$, the mean squared error of state estimation error is minimized by minimizing the trace of $P_{k}^{+}$. In other words, the trace of $P_{k}^{+}$ is differentiated with respect to $K_{k}$ and the result is set to zero in search of the minimizing conditions. That is

(A53) $$\begin{array}{c} \frac{\partial \mathrm{Tr}(P_{k}^{+})}{\partial K_{k}}=0=-2\left[I-K_{k}H_{k}\right]P_{k}^{-}H_{k}^{T}+2K_{k}\left[H_{k}\Sigma_{x,k}H_{k}^{T}+R_{k}+\Sigma_{y,k}\right] \end{array}$$

where the following trace identities are used

(A54) $$\begin{array}{c} \frac{\partial BAC}{\partial A}=B^{T}C^{T}\;\;\frac{\partial ABA^{T}}{\partial A}=A\left[B+B^{T}\right] \end{array}$$

Now, solving for $K_{k}$ in Equation (A53) gives

(A55) $$\begin{array}{c} K_{k}=P_{k}^{-}H_{k}^{T}{\left[H_{k}P_{k}^{-}H_{k}^{T}+H_{k}\Sigma_{x,k}H_{k}^{T}+R_{k}+\Sigma_{y,k}\right]}^{-1} \end{array}$$

where the matrix term with inverse, $\left[H_{k}P_{k}^{-}H_{k}^{T}+H_{k}\Sigma_{x,k}H_{k}^{T}+R_{k}+\Sigma_{y,k}\right]$, is the covariance of the innovation filter, $P_{zz,k}$.

Finally, substituting Equation (A55) into Equation (A52) yields,

(A56) $$\begin{array}{cc} P_{k}^{+} & =P_{k}^{-}-K_{k}H_{k}P_{k}^{-}-P_{k}^{-}H_{k}^{T}K_{k}^{T}+K_{k}{\left[H_{k}P_{k}^{-}H_{k}^{T}+H_{k}\Sigma_{x,k}H_{k}^{T}+R_{k}+\Sigma_{y,k}\right]}^{-1}K_{k}^{T}\\ \; & =P_{k}^{-}-K_{k}H_{k}P_{k}^{-}\\ P_{k}^{+} & =\left[I-K_{k}H_{k}\right]P_{k}^{-} \end{array}$$

Equation (A56) is the update equation for error covariance matrix with the optimal gain obtained in Equation (A55).

#### Appendix B.1.3. Key Observations

The linear quantized discrete-time Kalman filter (QDKF) closely resembles the standard Kalman filter equations. The round-off errors caused by state, measurement, and input quantization do not alter the filter structure. However, the round-off error variances become additive to the covariances in the propagation and the gain equations as shown in Table A2. It can be noticed from Equation (A55), the Kalman gain augments the round-off error covariances as weighting factors, reducing the applied gain and thereby amplifying covariance updates. This weighting in Kalman gain can be interpreted as optimally weighing the innovation into the state updates. Another observation is that if the model is receiving quantized input, these errors percolate into the covariance propagation step (Equation (A56)) which is a deviation from standard Kalman filter equations where the forcing input is typically assumed to be unaffected by noise. If there is no forcing input in the model dynamics, this input error covariance is disregarded. The resulting QDKF algorithm serves as an optimal state estimator, accommodating quantized states, measurements, and inputs while considering uncertainties in state and measurement evolution.

**Table A2:** Quantized discrete-time Kalman filter algorithm.

System Model	$x_{k+1}=\Phi_{k}\left(x_{k}+\epsilon_{x,k}\right)+\Gamma_{k}\left(u_{k}+\epsilon_{u,k}\right)+\gamma_{k}w_{k}$
Measurement Model	$y_{k}=H_{k}\left(x_{k}+\epsilon_{x,k}\right)+v_{k}+\epsilon_{y,k}$
Initialize	${\hat{x}}_{0}^{+}=E\left[x_{0}\right]$
	$P_{0}^{+}=E\left[\left({\hat{x}}_{0}-x_{0}\right){\left({\hat{x}}_{0}-x_{0}\right)}^{T}\right]$
State propagation	${\hat{x}}_{k+1}^{-}=\Phi_{k}{\hat{x}}_{k}^{+}+\Gamma_{k}u_{k}$
Covariance propagation	$P_{k+1}^{-}=\Phi_{k}\left(P_{k}^{+}+\Sigma_{x,k}\right)\Phi_{k}^{T}+\Gamma_{k}\Sigma_{u,k}\Gamma_{k}^{T}+\gamma_{k}Q_{k}\gamma_{k}^{T}$
Kalman gain	$K_{k}=P_{k}^{-}H_{k}^{T}{\left[H_{k}P_{k}^{-}H_{k}^{T}+H_{k}\Sigma_{x,k}H_{k}^{T}+R_{k}+\Sigma_{y,k}\right]}^{-1}$
State update	${\hat{x}}_{k}^{+}={\hat{x}}_{k}^{-}+K_{k}\left(y_{k}-H_{k}{\hat{x}}_{k}^{-}\right)$
Covariance update	$P_{k}^{+}=\left[I-K_{k}H_{k}\right]P_{k}^{-}$

## Appendix C. Quantized Square-Root Kalman Filter (QSRKF)

Theoretically, the discrete-time Kalman filter equations are adequate to achieve state estimates with minimum mean square error. For onboard finite-precision implementation, the Kalman filter equations are prone to numerical instability. Due to round-off errors, the propagation and update equations were found to compromise the symmetry and positive-definiteness of the covariance matrix often leading to negative variances in the estimation errors [2,4]. This degradation in the Kalman filter performance is addressed through numerically robust square-root Kalman filters. In fixed-point realization, the square-root Kalman filter helps reduce the dynamic range and mitigate the round-off errors by using matrix square-root operations. The square-root Kalman filter (SRKF) operates by computing the covariance propagation and update expressions in terms of square-root factors of the *a priori* and *a posteriori* covariance matrices. This requires taking matrix square-root of the state covariance matrix.

### Appendix C.1. Square-Root of State Covariance Matrix

The square-root of the state covariance matrix $P_{k}$ is given by $S_{k}$ and defined as:

(A57) $$P_{k}=S_{k}S_{k}^{T}$$

where the square-root factor $S_{k}$ can be computed using Cholesky factorization of the symmetric positive-definite $P_{k}$. Alternately, the square-root matrix can be efficiently computed using QR decomposition.

The QR algorithm decomposes a matrix $A\in R^{n\times m}$ into two factors: an orthogonal matrix $Q\in R^{n\times n}$, and an upper-triangular matrix $R\in R^{n\times m}$ such that A=QR, and m≥n. If $\tilde{R}\in R^{n\times n}$ is the upper triangular portion of R, then ${\tilde{R}}^{T}$ is the Cholesky factor of $P_{k}=AA^{T}$, i.e., ${\tilde{R}}^{T}=S_{k}$ such that ${\tilde{R}}^{T}\tilde{R}=AA^{T}$ [45]. Specifically, if $\tilde{R}=\mathrm{qr}{\left\{A^{T}\right\}}^{T}$, the qr{·} operation performs the QR decomposition and returns an upper-triangular portion of R only, then ${\tilde{R}}^{T}$ is the lower-triangular Cholesky factor of $P_{k}=AA^{T}$.

### Appendix C.2. Quantized Square-Root Kalman Filter for State Estimation

#### Appendix C.2.1. Initialization

As established, the SRKF is concerned with time and measurement update using square-root of the covariance matrices. As in the QDKF formulation (See Appendix B), the QSRKF filter is initialized with an initial mean $x_{0}^{+}$. However, in place of the state covariance matrix, the filter is initialized with its square-root $S_{0}^{+}$ calculated once using Cholesky factorization such that:

(A58) $$S_{0}^{+}=\sqrt{E[\left({\hat{x}}_{0}-x_{0}\right){\left({\hat{x}}_{0}-x_{0}\right)}^{T}]}$$

The filter description includes modeling quantization noise sources in the derivation along with the measurement and the process noises. The square-roots of the error covariances that correspond to the state, input, and measurement quantization noises (Equation (A43)), and the process, measurement noises (Equation (A38)) are calculated via Cholesky factorization. The square-root covariance matrices defined through the following notations:

(A59) $$\begin{array}{c} \Lambda_{x,k}=\sqrt{\Sigma_{x,k}}\;\Lambda_{y,k}=\sqrt{\Sigma_{y,k}}\;\Lambda_{u,k}=\sqrt{\Sigma_{u,k}}\;S_{w,k}=\sqrt{Q_{k}}\;S_{v,k}=\sqrt{R_{k}} \end{array}$$

#### Appendix C.2.2. Filter Propagation

In the propagation step, the time update for the state estimate remains unchanged:

(A60) $${\hat{x}}_{k+1}^{-}=\Phi_{k}{\hat{x}}_{k}^{+}+\Gamma_{k}u_{k}$$

The state covariance however must use only the matrix square-roots for propagation. The time-update for square-root of the state estimation error covariance $S_{k+1}^{-}$ is derived as

(A61) $$\begin{array}{cc} P_{k+1}^{-}= & \Phi_{k}P_{k}^{+}\Phi_{k}^{T}+\Phi_{k}\Sigma_{x,k}\Phi_{k}^{T}+\Gamma_{k}\Sigma_{u,k}\Gamma_{k}^{T}+\gamma_{k}Q_{k}\gamma_{k}^{T} \end{array}$$

(A62) $$\begin{array}{cc} S_{k+1}^{-}{\left(S_{k+1}^{-}\right)}^{T}= & \Phi_{k}S_{k}^{+}{\left(S_{k}^{+}\right)}^{T}\Phi_{k}^{T}+\Phi_{k}\Lambda_{x,k}\Lambda_{x,k}^{T}\Phi_{k}^{T}+\\ \; & \Gamma_{k}\Lambda_{u,k}\Lambda_{u,k}^{T}\Gamma_{k}^{T}+\gamma_{k}S_{w,k}S_{w,k}^{T}\gamma_{k}^{T} \end{array}$$

(A63) $$\begin{array}{cc} S_{k+1}^{-}{\left(S_{k+1}^{-}\right)}^{T}= & \left[\Phi_{k}S_{k}^{+}\;\;|\;\;\Phi_{k}\Lambda_{x,k}\;\;\left|\;\;\Gamma_{k}\Lambda_{u,k}\;\;\right|\;\;\gamma_{k}S_{w,k}\right]{\left[\Phi_{k}S_{k}^{+}\;\;|\;\;\Phi_{k}\Lambda_{x,k}\;\;\left|\;\;\Gamma_{k}\Lambda_{u,k}\;\;\right|\;\;\gamma_{k}S_{w,k}\right]}^{T} \end{array}$$

(A64) $$\begin{array}{cc} S_{k+1}^{-}= & \mathrm{qr}{\left\{{\left[\Phi_{k}S_{k}^{+}\;\;|\;\;\Phi_{k}\Lambda_{x,k}\;\;\left|\;\;\Gamma_{k}\Lambda_{u,k}\;\;\right|\;\;\gamma_{k}S_{w,k}\right]}^{T}\right\}}^{T} \end{array}$$

#### Appendix C.2.3. Kalman Gain

To compute the Kalman gain for the QSRKF, the expressions for the square-root of the innovation covariance is first developed. The innovation covariance $P_{zz}$ of the QDKF algorithm (Equation (A55)) is given as:

(A65) $$P_{zz,k}=H_{k}P_{k}^{-}H_{k}^{T}+H_{k}\Sigma_{x,k}H_{k}^{T}+R_{k}+\Sigma_{y,k}$$

Similar to the propagation step, the square-root factors for the innovation covariance is developed as:

(A66) $$\begin{array}{cc} S_{zz,k}S_{zz,k}^{T} & =H_{k}S_{k}^{-}{\left(S_{k}^{-}\right)}^{T}H_{k}^{T}+H_{k}\Lambda_{x,k}\Lambda_{x,k}^{T}H_{k}^{T}+S_{v,k}S_{v,k}^{T}+\Lambda_{y,k}\Lambda_{y,k}^{T} \end{array}$$

(A67) $$\begin{array}{cc} S_{zz,k} & =\mathrm{qr}{\left\{{\left[H_{k}S_{k}^{-}\;\;|\;\;H_{k}\Lambda_{x,k}\;\;\left|\;\;S_{v,k}\;\;\right|\;\;\Lambda_{y,k}\right]}^{T}\right\}}^{T} \end{array}$$

The Kalman gain $K_{k}$ of the QDKF method is known to be $P_{k}^{-}H_{k}^{T}{\left(P_{zz,k}\right)}^{-1}$. Using the calculated square-root innovation covariance factor, the Kalman gain is computed as:

(A68) $$\begin{array}{c} K_{k}=S_{k}^{-}{\left(H_{k}S_{k}^{-}\right)}^{T}{\left(S_{zz,k}S_{zz,k}^{T}\right)}^{-1} \end{array}$$

#### Appendix C.2.4. Filter Update

The state and the state covariance update equations are computed using the Kalman gain presented in Equation (A68). Starting from the symmetric version of the covariance update equation (Equation (50)), the square-root factored form of the covariance update, $S_{k}^{+}$, is computed as:

(A69) $$\begin{array}{cc} P_{k}^{+} & =\left[I-K_{k}H_{k}\right]P_{k}^{-}{\left[I-K_{k}H_{k}\right]}^{T}+K_{k}\left[H_{k}\Sigma_{x,k}H_{k}^{T}+R_{k}+\Sigma_{y,k}\right]K_{k}^{T} \end{array}$$

(A70) $$\begin{array}{cc} S_{k}^{+}{\left(S_{k}^{+}\right)}^{T} & =\left[I-K_{k}H_{k}\right]S_{k}^{-}{\left(S_{k}^{-}\right)}^{T}{\left[I-K_{k}H_{k}\right]}^{T}+\\ \; & \;\;K_{k}\left[H_{k}\Lambda_{x,k}\Lambda_{x,k}^{T}H_{k}^{T}+S_{v,k}S_{v,k}^{T}+\Lambda_{y,k}\Lambda_{y,k}^{T}\right]K_{k}^{T} \end{array}$$

(A71) $$\begin{array}{cc} S_{k}^{+} & =\mathrm{qr}\{{\left[\left[I-K_{k}H_{k}\right]S_{k}^{-}\;\;|\;\;K_{k}\left[H_{k}\Lambda_{x,k}\;\;\left|\;\;S_{v,k}\;\;\right|\;\;\Lambda_{y,k}\right]\right]}^{T}{\}}^{T} \end{array}$$

**Table A3:** Quantized square-root Kalman filter algorithm.

System Model	$x_{k+1}=\Phi_{k}\left(x_{k}+\epsilon_{x,k}\right)+\Gamma_{k}\left(u_{k}+\epsilon_{u,k}\right)+\gamma_{k}w_{k}$
Measurement Model	$y_{k}=H_{k}\left(x_{k}+\epsilon_{x,k}\right)+v_{k}+\epsilon_{y,k}$
Initialize	${\hat{x}}_{0}^{+}=E\left[x_{0}\right]$
	$S_{0}^{+}=\sqrt{E[\left({\hat{x}}_{0}-x_{0}\right){\left({\hat{x}}_{0}-x_{0}\right)}^{T}]}$
State propagation	${\hat{x}}_{k+1}^{-}=\Phi_{k}{\hat{x}}_{k}^{+}+\Gamma_{k}u_{k}$
Square-root covariance propagation	$S_{k+1}^{-}=\mathrm{qr}{\left\{{\left[\Phi_{k}S_{k}^{+}\;\;|\;\;\Phi_{k}\Lambda_{x,k}\;\;\left|\;\;\Gamma_{k}\Lambda_{u,k}\;\;\right|\;\;\gamma_{k}S_{w,k}\right]}^{T}\right\}}^{T}$
Innovation Covariance	$S_{zz,k}=\mathrm{qr}{\left\{{\left[H_{k}S_{k}^{-}\;\;|\;\;H_{k}\Lambda_{x,k}\;\;\left|\;\;S_{v,k}\;\;\right|\;\;\Lambda_{y,k}\right]}^{T}\right\}}^{T}$
Kalman gain	$K_{k}=S_{k}^{-}{\left(H_{k}S_{k}^{-}\right)}^{T}{\left(S_{zz,k}S_{zz,k}^{T}\right)}^{-1}$
State update	${\hat{x}}_{k}^{+}={\hat{x}}_{k}^{-}+K_{k}\left(y_{k}-H_{k}{\hat{x}}_{k}^{-}\right)$
Square-root covariance update	$S_{k}^{+}=\mathrm{qr}\{{\left[\left[I-K_{k}H_{k}\right]S_{k}^{-}\;\;|\;\;K_{k}\left[H_{k}\Lambda_{x,k}\;\;\left|\;\;S_{v,k}\;\;\right|\;\;\Lambda_{y,k}\right]\right]}^{T}{\}}^{T}$
